# Food and nutrition security under global trade: a relation-driven agent-based global trade model

**DOI:** 10.1098/rsos.201587

**Published:** 2021-01-13

**Authors:** Jiaqi Ge, J. Gareth Polhill, Jennie I. Macdiarmid, Nuala Fitton, Pete Smith, Heather Clark, Terry Dawson, Mukta Aphale

**Affiliations:** 1 School of Geography, University of Leeds, Leeds LS2 9JT, UK; 2 Information and Computational Science, The James Hutton Institute, UK; 3 The Rowett Institute, University of Aberdeen, Aberdeen AB24 3FX, UK; 4 School of Biological Sciences, University of Aberdeen, Aberdeen AB24 3FX, UK; 5 The Institute of Applied Health Sciences, University of Aberdeen, Aberdeen AB24 3FX, UK; 6 Department of Geography, King's College London, UK

**Keywords:** global trade, relation-driven trade, food security, micronutrients, agent-based model

## Abstract

This paper addresses the highly relevant and timely issues of global trade and food security by developing an empirically grounded, relation-driven agent-based global trade model. Contrary to most price-driven trade models in the literature, the relation-driven agent-based global trade model focuses on the role of relational factors such as trust, familiarity, trade history and conflicts in countries' trade behaviour. Moreover, the global trade model is linked to a comprehensive nutrition formula to investigate the impact of trade on food and nutrition security, including macro and micronutrients. Preliminary results show that global trade improves the food and nutrition security of countries in Africa, Asia and Latin America. Trade also promotes a healthier and more balanced diet, as countries have access to an increased variety of food. The effect of trade in enhancing nutrition security, with an adequate supply of macro and micronutrients, is universal across nutrients and countries. As researchers call for a holistic and multifactorial approach to food security and climate change (Hammond and Dubé 2012 *Proc. Natl Acad. Sci. USA* **109**, 12 356–12 363. (doi:10.1073/pnas.0913003109)), the paper is one of the first to develop an integrated framework that consists of socio-economic, geopolitical, nutrition, environmental and agri-food systems to tackle these global challenges. Given the ongoing events of Brexit, the US–China trade war and the global COVID-19 pandemic, the paper will provide valuable insights on the role of trade in improving the food and nutrition security across countries.

## 1. Background

In 2019, 821.6 million people in the world are hungry and 2 billion people (26.4% of the world's population) are food and nutrition insecure, who lack access to safe, sufficient and nutritious food, according to the State of Food Security and Nutrition [1]. Despite great progress in agri-technology in the last century, hunger is increasing in poor countries, especially during economic slowdowns and downturns. In addition to the degradation of ecosystems and more frequent crop failures due to climate change [2], recent trade disputes and conflicts among countries, and the outbreak of the COVID-19 pandemic have all posed threat to global food supply and food security [3]. As a result, the world is struggling to meet the WHO nutrition target by 2025 or the UN sustainable development goal's nutrition target by 2030.

Food production and supply concerns more than the agri-food systems. They are deeply coupled with the social, economic and geopolitical systems at both local and global scales. Using a large-scale simulation model of land-use change, Brown *et al*. [4] show that social and behavioural factors can drastically change local land use and cause severe food shortages of up to 56% without climatic disturbances. Hammond and Dubé [5] argue that food and nutrition security is driven by complex underlying systems at both local and regional/global scales. The authors call for a systems approach using transdisciplinary modelling tools such as system dynamics and agent-based modelling.

One important mechanism for food allocation is via trade. In 2013, about 23% of the food produced for human consumption was traded internationally [6], which feeds 2–3 billion people globally and uses 13% of worldwide cropland and pasture [7,8] and the proportion continues to grow annually. As globalization deepens, spatially distant countries become increasingly connected by trade, and so are their agri-food systems, a phenomenon called ‘telecoupling’. For example, Challies *et al*. [9] show how surging pork demand in Germany caused large-scale deforestation in Brazil, as the latter is a major supplier of soya beans, which are used as pig feed in Germany [9]. Similarly, Fuchs *et al*. [3] discuss how the recent US–China trade war can cause large-scale deforestation in the Amazon, as China switched to Brazil for soya imports. Disputes and conflicts between any two countries can thus lead to a serious disturbance to global trade and food security.

### 1.1. Price versus relation-driven trade models

Conventional trade models are based on the general equilibrium (GE) theory in economics, where trade volumes are expressed as mathematical equations of commodity prices. Solutions to the equations are found (analytically or numerically) by adjusting the prices so that supply equals demand (market clearing) in every sector. One of the common methods to derive numerical solutions is computable general equilibrium (CGE), which is used in models such as global trade analysis project (GTAP) [10]. CGE trade models have been used to evaluate the impact of trade policies such as tariffs and trade liberalization (e.g. [11]). Apart from GE models, partial equilibrium (PE) models are also developed for the agricultural sector, upon which integrated assessment models such as common agricultural policy regionalized impact model (CAPRI) [12] are based. Both CGE and PE models are built upon the key assumptions of equilibrium, price elasticities and the market-clearing condition. However, these theoretical frameworks offer limited possibilities for rigorous testing against historical data and experience [13].

The gravity model of trade, on the other hand, takes an empirical approach, which assumes that trade volume between two countries is proportional to the GDP of the two countries and disproportional to their geographic distance [14,15]. The model has since become one of the most successful empirical models in economics [16,17]. A price mechanism and market clearing, which are central in CGE and most macroeconomic trade theories, are not part of the gravity trade model. There is thus a gap between CGE model, which is based on theory but lacks predictive power, and the gravity model, which has predictive power but lacks theoretical underpinning.

What conventional price-driven trade models like CGE do not consider is other relational factors such as trust, familiarity, conflicts and competition in a trade relationship. For example, evidence shows that countries that share culture, language, religion and institutional structure trade more even after controlling for geographic distance and GDP [18–25]. Trust and familiarity play an important role in trade relationships. Using trade data of 25 countries, Den Butter and Mosch [26] show that trust explains a large fraction of trade volume between any two countries by reducing transaction costs. Deardorff [27] argues that increasing trust and reducing unfamiliarity is crucial in establishing new trade relationships, especially for countries culturally high in uncertainty aversion. Huang [28] shows empirically that transport cost is not the only reason that distant countries trade less. Countries trade also less because they have never traded before and are thus unfamiliar with each other.

For perishable goods such as food and agricultural products, trust, reputation and previous trade relationship is particularly important. Because the conditions of perishable goods are hard to enforce by contract compared with manufactured goods, importers and exporters rely on mutual trust and repeated transactions to assure contractual performance. Macchiavello and Morjaria [29] show the value of long-term relationships based on trust and reputation in rose exports in Kenya. They find that when supply disturbances occur, exporters have incentives to prioritize delivery to buyers with whom they have traded before to protect their reputation. Similarly, research has found that trust, reputation and a previous trade relationship are particularly important for perishable and non-enforceable goods such as foods and agricultural products [30,31].

Trade can also be driven by geopolitical conflicts and competition among countries. For example, China's decision to buy soya from Brazil and not the US is driven by its relationship with the US rather than any ‘rational’ economic factors. Similarly, Brazil uses soya bean exports to secure land ownership in neighbouring countries, particularly Paraguay and Bolivia, extend political influence in Africa and balance trade with China [32]. Countries may also engage in (irrational) competition with each other for essential foods, especially in a crisis. Timmer [33] shows how, in the 2008 rice crises, countries in Southeast Asia competed to secure rice by hoarding and banning exports, causing spikes in rice price far exceeding what classic economic theory would suggest based on the initial (moderate) fall in supply. Timmer [34] concludes that complex human behaviours, such as loss aversion, time inconsistency and herd behaviour can present significant challenges to a traditional ‘economic optimization’ approach to trade.

In summary, existing price-driven global trade models are insufficient to fully capture trade relationships among countries. Relational factors such as trust, familiarity, conflicts and competition are just as important. When trust and a previous trade relationship is needed for trade to happen, countries will miss potential trade opportunities if they don't have trust between them [26], leaving the market to not clear in reality. We thus need a relation-driven approach to global trade to complement the price-driven one, which this paper will develop.

### 1.2. Countries as agents

Although trade activities are carried out by individual firms, most economic global trade models such as CGE and gravity models treat countries as the entity of trade. Similarly, many agent-based models (ABMs) of trade and international relations also treat countries as encapsulated agents that interact with each other [22,35,36]. In fact, the question ‘what constitutes an agent’ remains open in the ABM community. Macal and North [37] summarize four properties most ‘agents’ in ABM have: (i) autonomy (function independently in their environment), (ii) modularity (identifiable, discrete entity), (iii) sociality (interact with other agents), and (iv) conditionality (have a state that varies over time). Entities that meet the above four criteria have been represented as agents in the ABM literature, ranging from a biological cell to a person to an organization to a country.

Conceptually, it is appropriate to treat countries as agents if the model aims to study country-wise trade relationships, because a country makes decisions on national trade policies and positions, and behaves as a single entity as it sets up trade barriers, joins trade agreements and engages in trade wars [34,35]. Moreover, a country shares common attributes such as GDP per capita, institutional structure, languages and culture, so it can be treated as a single agent when researchers look at the role of these attributes in trade [22,23]. On the other hand, treating countries as a single unit misses the complex behaviour of and interactions between individual firms that carry out the actual trade. Treating countries as agents also miss the internal trade flows between regions in a country. Hence more other studies have been using firm-level and/or regional-level data to study heterogeneous firm behaviour in trade (e.g. [29]) and intra-national trade (e.g. [38]).

Practically, however, most global trade data are available at the country level, including the ones from the UN and OECD, which we use in this study. Although some advanced countries have much more detailed trade data at the regional level, such segregated data are not available for all countries in the world, especially the lower income ones that are more prone to food insecurity. Even more scarce are firm-level transaction data, which are often exclusive, almost always incomplete and vary greatly by countries and sectors. The lack and incompleteness of empirical data at lower than the country level severely restricts the types of empirically grounded agent-based model researchers can develop, especially if the model aims to include all countries and sectors. Finally, as Ge and Polhill [39] have argued, agent representation is rather a narrative concept, and the appropriate agent will depend on the aims and purposes of the study, the research questions, and the constraints imposed by data, ontological complexity and computational capacity.

### 1.3. Multivariate nutrition

Most research on food security has so far focused on energy consumption (calories). However, having sufficient energy does not guarantee a nutritionally adequate diet. While obesity becomes a problem even in the poorest parts of the world [40], nutrient deficiency, especially deficiency in micronutrients, are still prevalent in both low- and high-income countries [41]. For example, more than 2 billion people in the world are deficient in iron; 21% of children are deficient in vitamin A, which is the direct cause of 800 000 deaths per year [42]. In 1996, the Food and Agriculture Organization (FAO) amplified the definition of food security to include a sufficient supply of nutrients in the diet. The UN's sustainable development goal (SDG Target 2) ‘Zero Hunger’ aims to meet the nutritional needs of all people, especially those susceptible to micronutrient deficiency (adolescent girls, pregnant and lactating women and older persons).

Previously the majority of food security research has focused on staple foods that are the main sources of calories, such as wheat, rice, soya beans and maize (e.g. [43–45]), although more recent studies have been looking at a larger variety of foods (e.g. [46–48]). To achieve food and nutrition security by FAO and SDG 2 standards, however, one needs a diverse, balanced diet containing a variety of foods, such as those rich in vitamin A (offal, oranges, carrots), iron (e.g. red meat, offal, spinach) and zinc (e.g. meat, seafood, nuts).

This paper develops an empirically grounded, relation-driven agent-based global trade model to study the impact of global trade on food and nutrition security of countries across the world. It addresses the highly relevant and timely issues of trade and food security given the current debates about trade agreements associated with Brexit and US–China trade dispute. The paper makes several important contributions to the current literature. First, it will develop a relation-driven global trade model to complement the price-driven trade models that dominate the literature. It will provide a more flexible framework to incorporate more complex, relation-driven trade behaviour of countries. The model developed in this paper can thus enhance our understanding of some trade phenomenon such as repeated trade, preferential treatment in trade and trade conflicts, which are common in the real world but hard to explain using price-driven trade theories. It also relaxes the restrictive assumptions in CGE models, including market-clearing and equilibrium condition and price-driven trade behaviour. Second, the trade model will include a comprehensive list of foods and be linked to a nutrition formula based on food consumption to investigate the impact of trade on food and nutrition security. The model can thus be used to identify countries and regions most vulnerable to food and nutrition shortage, and which macro- and micro-nutrition they are likely to lack under different scenarios.

## 2. Methods

This paper describes the development of an empirical agent-based model of global food trade to study the impact of trade and climate change on food and nutrition security of countries across the world. The empirical agent-based model is implemented in NetLogo [49]. In this section, we will follow the guidelines of Grimm *et al*.'s overview, design concepts and details (ODD) protocol [50,51] to describe the model.

### 2.1. Purpose

The purpose of the model is to study the impact of global food trade on food and nutrition security in countries around the world. It will incorporate three main aspects of trade between countries, including a country's wealth, geographic location and its trade relationships with other countries (past and ongoing), and will be used to study food and nutrition security across countries in various scenarios, such as climate change, sustainable intensification, waste reduction and dietary change.

### 2.2. Agent classes and attributes

#### 2.2.1. Countries

As previously discussed, for both conceptual and practical reasons, we choose to represent countries as agents, which we believe is the most suitable for the study and the research questions we will address, as well as the most practical given the data and computational constraints. Each country in the model has a list of attributes (table 1), including geographic location, population size, GDP, production of (multi-dimensional) food commodities, the country's historic trade relationships with other countries and so on. We include the 165 countries in the world for which complete data of the food supply from the FAO food balance sheet are available. The model is spatially explicit in that the countries are represented spatially at a global scale.

**Table 1.** Selected attributes of a country agent.

variable	description	data source (if exogenous)	En?^a^	D?^b^
country name	name of the country, including variations	FAO	N	N
location	country location in a global map	GIS	N	N
area	geographic area	GIS	N	N
region and sub-region	the region and sub-region a country belongs to	UN	N	N
initial GDP	In 2000	FAO	N	N
initial population	In 2000	FAO	N	N
initial production	tonnes of each food commodity produced in the country in 2000	FBS	N	N
initial import	tonnes of each commodity imported from all other countries in 2000	FBS	N	N
initial export	tonnes of each commodity exported from this country to all other countries in 2000	FBS	N	N
initial domestic supply (DS)	DS of all commodities in the country in 2000 (DS = production + import – export – stock = food + feed + processing + loss)	FBS	N	N
percentage of food in DS	percentage of food in DS for each commodity in 2000 (food/DS)	derived from FBS	N	N
percentage of loss in DS	percentage of loss in DS for each commodity in 2000 (loss/DS)	derived from FBS	N	N
is intermediary^c^	whether a country is a trade intermediary for each commodity	derived from FBS	N	N
import needed—domestic	import needed for domestic consumption for each commodity	endogenous	Y	Y
import needed—intermediary	import needed for re-export for each commodity (only relevant for intermediaries for the commodity)	endogenous	Y	Y
export available	export available for each commodity	endogenous	Y	Y
import realized	import (both domestic and intermediary) realized in each commodity	endogenous	Y	Y
export realized	export (both domestic and intermediary) realized in each commodity	endogenous	Y	Y
current food supply	food supply of each commodity in the current year	endogenous	Y	Y
current GDP	GDP in the current year	FAO if year ≤ 2013; OECD projection if year >2013		
current population	population in the current year	FAO if year ≤ 2013; OECD projection if year > 2013	N	Y
current production	production of each commodity in the current year	FBS if year ≤ 2013; Scenario projection if year > 2013	N	Y
typical diet^c^	the country's food supply of each commodity in 2000	FBS	Y	Y
fortification	how the country fortifies its wheat products	food fortification initiative http://www.ffinetwork.org/index.html	N	N
household waste^c^	the percentage of household food wasted for each commodity	Gustavsson *et al*. [52]	N	N
population-level nutrient required per person^c^	population-level nutrient required per person in the given year in macro and micronutrients based on the demographic composition	WHO recommendations	N	Y
current nutrient supply consumed per person	average nutrient supply per person in the given year in macro and micronutrient	endogenous, calculated from food consumption	Y	Y

^a^If the variable is endogenous (same for all tables in this section).

^b^If the variable is dynamic or will change over time (same for all tables in this section).

^c^More explanations below.

The activities country agents engage in are the production, trade and food intake, which is a multi-dimensional variable consisting of 91 food commodities, consistent with those used in FAO food balance sheets (FBS) (see §2.4.3). The production of food commodities in each country is exogenous and changes every year depending on the scenarios. Once the production for the year is revealed or completed, countries trade with each other if they have unfulfilled domestic demand or unconsumed domestic supply. A country's food supply is then a combined outcome of its domestic production and trade with other countries. We do not consider inequalities within a country in access to food, which can be great in some countries. In this study, we focus on the average food intake per capita, which we use to compare with food requirement per capita, as an indicator of a country's food and nutrition security. Indicators of country-specific inequality (such as the GINI index) can be built into the model later on, which is beyond the scope of this study.

Table 1 lists selected attributes of a country agent.

##### 2.2.1.1. Trade intermediary

For a given commodity, an intermediary country is one that imports food for the purpose of re-exporting. As discussed before, intermediary countries are important facilitators of trade. In the model, we define a country as an intermediary for the commodity if the total export of the commodity is more than 80% of the total import (i.e. the majority of import is for re-exporting) in 2000, the baseline year. The motivation and trade behaviour of an intermediary country will be different from other non-intermediary countries that import for domestic consumption.

##### 2.2.1.2. Typical diet

We use the term ‘diet’ in the paper, but this is based on the national supply of food taken from the FAO food balance sheets (FBS), adjusted by the proportion that is inedible (e.g. banana peels) and wasted, which differs by region. Typical diet varies across countries and reflects a country's tradition and culture, as well as their natural and land-use conditions. When considering nutrient sufficiency and dietary change, we need to make sure that we do not naively prescribe countries an ‘ideal diet’ (nutritionally adequate) that is unrealistic to implement. In the model, we use the average reported food consumption between 2000 and 2002 (to smooth out fluctuations in any one year) as the baseline for each country's typical diet. The food composition in the typical diet changes every year in proportion to global food production and supply, to reflect the fact that the diet of people changes gradually (not drastically) over time. We assume that a country will aim to obtain the typical diet for its population in the current year; it will import a food commodity if it produces less domestically than is needed in the typical diet and export if it produces more. Some countries may fail to feed their populations with the typical diet; nor does a country's typical diet necessarily guarantee nutrient sufficiency, which reflects the situations in reality.

##### 2.2.1.3. Fortification

In some countries where wheat is refined and stripped of fibre and micronutrients, the flour and refined cereals are fortified replacing some of the micronutrients. The most commonly fortified food is wheat (flour) [53]. Hence, when calculating nutrient supply for each country, we need to adjust for the nutrient content of wheat in each country depending on if it is refined or not, then if the refined flour is fortified. This varies by the income of countries, with higher income countries more commonly refining than fortifying with micronutrients. The level of fortification also varies by country. The refined flour and cereal, however, will have a lower supply of fibre. Food composition values are derived from the USDA food composition database (2014, release 27) [54]. Food Fortification Initiative specifies whether food is fortified or not in a country, and the income levels for countries are as specified by the World Bank.^1^

##### 2.2.1.4. Household waste

Food waste up to the point of the household is accounted for in the FBS, but not waste generated in the household, after production and trade where a certain percentage of food will be wasted. Not accounting for household waste will lead to an overestimation of nutritional intake based on food consumption. The amount of food wasted depends on the type of food and the countries and regions. Generally speaking, countries and regions that are wealthier waste more food at the household level. Note that household waste does not include the part of food that is inedible, such as banana peel, which has already been accounted for in the nutrient calculation. Table 2 shows the percentage of food waste, based on food that could have been eaten, in household consumption by region and food type, which is estimated in Gustavsson *et al*. [52].

**Table 2.** Percentage of food waste in household consumption.

region	*cereals*	*roots & tubers*	*oilseeds*	*pulses*	*nuts*	*fruit*	*veg*	*meat*	*offal*	*fish*	*milk*
Europe	*25*	*17*	*4*	*4*	*4*	*19*	*19*	*11*	*11*	*11*	*7*
North America, Oceania	*27*	*30*	*4*	*4*	*4*	*28*	*28*	*11*	*11*	*33*	*15*
industrialized Asia	*20*	*10*	*4*	*4*	*4*	*15*	*15*	*8*	*8*	*8*	*5*
sub-Saharan Africa	1	2	1	1	1	5	5	2	2	2	0.1
North Africa, West and Central Asia	12	6	2	2	2	12	12	8	8	4	2
South and Southeast Asia	3	3	1	1	1	7	7	4	4	2	1
Latin America	10	4	2	2	2	10	10	6	6	4	4

##### 2.2.1.5. Nutrient requirements

Because people in different gender–age groups have different nutritional needs, the population-level nutrient requirement per person in a country is based on the demographic composition of its population. Countries with a larger young adult and male population will have a higher nutrient requirement than those with an ageing population. The demographic composition of a country will change over time, and so will the population-level nutrient requirement. An adequate energy intake was estimated using population-weighted average dietary energy requirements (ADERs), calculated using data for each age and sex with assumptions of physical activity level (PAL) being 1.75 and BMI being 21 kg m^−2^ for adults. The population-level nutrient requirement of a country will be compared with the nutrient supply in the country in any given year to determine a country's level of nutrient sufficiency. The nutrient requirements are from the WHO [55,56].

#### 2.2.2. Trade relationships between countries

As previously said, the trade model will be relation-driven, which means that trade decisions will depend on previous bilateral trade relationships between countries as they engage in trade repeatedly over time, in addition to their geographic location and ability to pay. We assume countries rank each other with different trade priorities to determine whom to trade with (assuming there are multiple competing buyers or sellers). Trade priority will depend on four elements: (i) GDP per capita, (ii) geographic distance, (iii) historic trade relationship, and (iv) emergent trade relationship.

The first element, GDP per capita, serves as a proxy for a country's ability to pay for a commodity. Priority is given to countries with a higher GDP per capita or high ability to pay. The second element, geographic distance between the two trading countries, is an important factor in predicting trade volumes: countries close to each other tend to trade more. One reason is the lower transport cost. Another reason is that countries close to each other are also more familiar with each other, and more likely to have a similar culture, customs and languages, all of which facilitate trade [28].

The third element, historic trade relationship, is measured by trade volume of all commodities in the year 2000. We use trade volume as an indicator of the existing trade relationships established between the two countries. As research has shown, countries that are more familiar with each other (via common language, religion, institutional structure and other social and cultural characteristics) trade more often [19,20,57]. While geographic proximity is one cause for enhanced familiarity and trust between countries, there are other non-geographic factors, such as historic connections (e.g. former colony, commonwealth) [58] and international organizations or trade unions (e.g. OECD, EEA, Trans-Pacific Partnership), that could cause some countries to have closer connections and thus trade more. Historic trade volume will reflect these non-GDP and non-geographic factors. We distinguish trade volumes by exports and imports because they represent different roles in trade relationships.

The last element, emergent trade relationship, allows new trade to emerge endogenously and to influence subsequent trade development in a path-dependent way. While the first three elements are exogenous to the model and deterministic, the last element is endogenous and stochastic. Trade relationship that evolved from the model has an impact on future trade decisions. Two countries low on each other's trade priority (due to low GDP per capita, long geographic distance or few trade records before) can start a new trade relationship in a year when they fail to trade with their usual partners (e.g. due to crop failure or market disturbances). Such an ‘incidental’ new trade between the two countries will increase their ranks in each other's trade priority, which will in turn increase the chances that they trade again in the future.

The trade priority (for importing and exporting partners) assigned by countries to one another is a weighted average of the four elements above. The weights are calibrated using actual trade and consumption data. We allow the weight to be zero in the search space, so that if any of the above elements do not have a significant impact on trade patterns in the empirical data, it will not have an effect in the model either. Although the second and third elements may be correlated (e.g. many countries that trade often are also geographically close), they do not coincide. For example, countries that have developed historical trade links may not be geographically close, such as the commonwealth countries; on the other hand, geographically close countries may not trade as much, such as the US and Cuba. Hence, we should still be able to distinguish the effect of the four elements when calibrating the weights for the elements using empirical data.

In this model, we do not try to emulate price dynamics or predict future prices. Although prices are not explicitly modelled, the price mechanism to allocate commodities among countries will be partially incorporated in the ranking and matching process of trade partners through countries' GDP per capita, geographic location, production, consumption and dietary preferences. A country's GDP per capita and dietary preference determine its purchasing power and willingness to pay for a product. Moreover, a country's production reflects its overall productivity and production costs of the food; its consumption reveals a country's budget, dietary habits and preferences; its location is a proxy for transportation costs. Therefore, although the model does not include the price mechanism directly, it does implicitly incorporate the information that prices contain.

Table 3 shows the attributes of the trade relationship class.

**Table 3.** Attributes of a trade relationship.

variable	data source if not endogenous	En?^a^	D?^b^
GDP per capita	FAO (year <=2013); OECD projection (year > 2013)	N	Y
distance	GIS	N	N
historic trade relationship—import	FAO FBS data for years 2000–2002	N	N
historic trade relationship—export	FAO FBS data for years 2000–2002	N	N
emergent trade relationship—import		Y	Y
emergent trade relationship—import		Y	Y

^a^If the variable is endogenous (same for all tables in this section).

^b^If the variable is dynamic or will change over time (same for all tables in this section).

#### 2.2.3. Food and nutrients

As was discussed before, we must look at a more comprehensive food list than a few major crops to gain a better understanding of the nutrient sufficiency, especially micronutrient sufficiency across countries. In this study, we include 91 food categories as in FAO's FBS. Countries will produce, trade and consume a different amount in each food category, which we then use to calculate macro and micronutrients. The aggregated groups to which each food category belongs are as follows: ‘Cereals – Excluding Beer’, ‘Starchy Roots’, ‘Sugar Crops’, ‘Sugar & Sweeteners’, ‘Pulses’, ‘Treenuts’, ‘Oilcrops’, ‘Vegetable Oils’, ‘Vegetables’, ‘Fruits – Excluding Wine’, ‘Spices’, ‘Stimulants’, ‘Alcoholic Beverages’, ‘Meat’, ‘Offal’, ‘Animal fats’, ‘Eggs’, ‘Milk – Excluding Butter’, ‘Fish; Seafood’, ‘Aquatic Products’ and ‘Other (e.g. Infant food and miscellaneous)’.

The mapping of food to nutrients is based on data from the GENuS project [59]. For a detailed description of the methodology see [46]. Each of the 91 food categories, which are made up of many food items, was disaggregated to individual food items. Each food commodity was then mapped to the nutrient composition. A weighted mean of the nutrient composition for the food items was used when the data were aggregated back to the food commodity groups. This was based on the global production of each food item within that group (FAO production data). If there were no production data for food items in a food commodity, an unweighted average was used. The nutrient data came primarily from the USDA food composition data.^2^ If any food item was not in the USDA tables, the composition was taken from other regional food composition tables. In this study, we exclude the nutrients for which the mapping involves large uncertainties, leaving the following: calories (energy, kcal), protein, fat, vitamin C, vitamin A, folate, calcium, iron, zinc, dietary fibre, thiamin, riboflavin, niacin, vitamin B6 and saturated fat.

### 2.3. Process overview and scheduling

Each step in the simulation represents a year in real time. We choose an annual time step because it is appropriate for the production cycle and the time-scale of the model (2000–2050), and also because data on trade, production, GDP and population are only available annually. The starting year of the model was 2000, which is also the baseline year.

At the beginning of each year, countries receive the production of all food from last year, which is the amount available for domestic consumption and trade. If a country has produced more than it needs for domestic consumption of the typical diet, it will try to export the excess; if a country has produced less than it needs for domestic consumption, it will try to import the deficiency. Also, if a country is an intermediary of a food commodity, it will want to import food for re-export, even though it may have a sufficient amount for domestic consumption.

**Figure 1.**
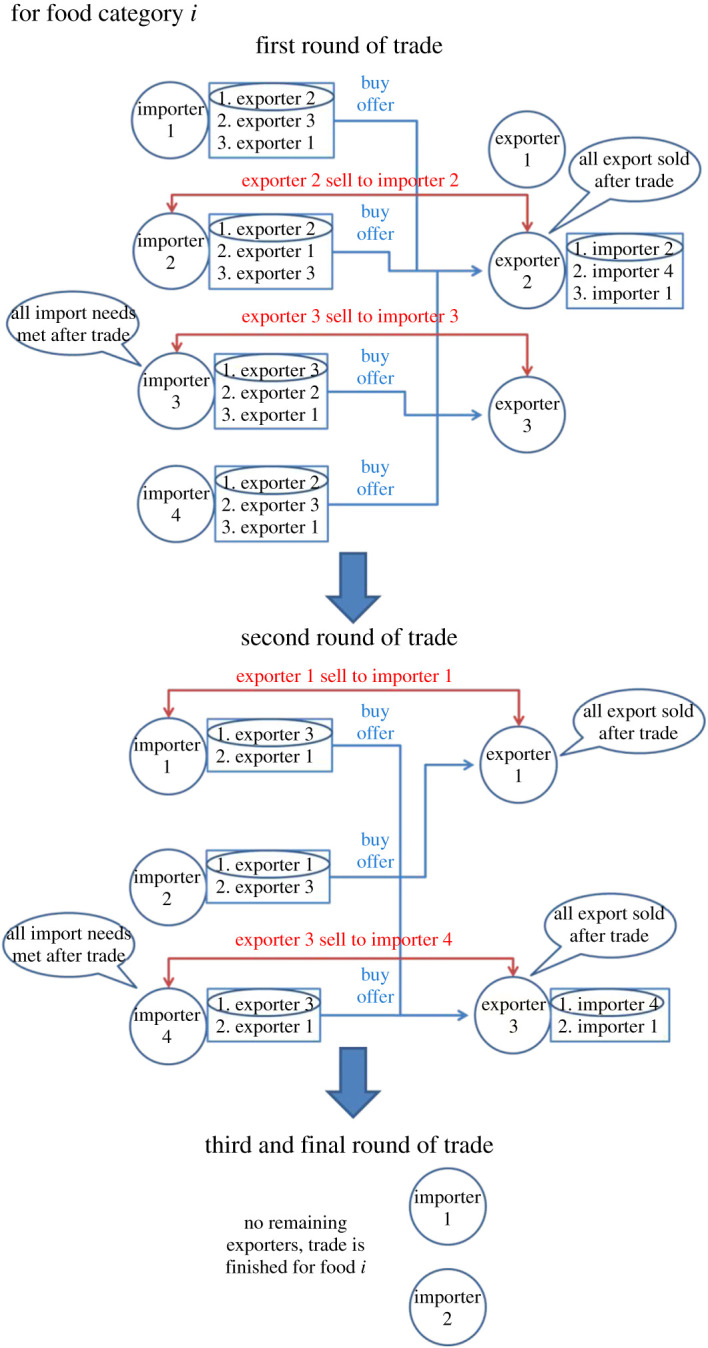
The trade procedure: the ranking and matching of importers and exporters.

After countries decide on their trade positions (whether to import or export, and the amount to import or export), they will start looking for trade partners. Importers will first send buying offers to potential exporters. If there are multiple exporters of the food on the market, importers will send the offer to the country with the highest trade priority on their lists (see §2.2.2). After importers have sent offers, exporters will decide to whom they will sell their commodity, if they receive multiple buying offers. Similarly, exporters will evaluate importers' priorities and sell only to the ones with the highest priority until all the available food for export is sold. Importers whose buying offers are not satisfied in this round will repeat the same process in the next round until their import demand is satisfied or there is nothing left to buy. To prevent some large countries with high demand from flooding and dominating the market, we limit each trade transaction to a maximum amount, so that smaller countries can compete with larger countries. We emphasize that it is not the maximum amount a country can trade in each commodity in total; it is the maximum amount a country can trade in each commodity *per round*, and the trade of a commodity can take several rounds (on average 5–20) to finish (figure 1). Hence, countries with a large demand will try to buy again in subsequent rounds until their demand is fulfilled. We have conducted a sensitivity analysis using different values for the maximum amount per round, and the results are not sensitive to the parameter.

It will take several rounds for the market to clear and all the trade opportunities are realized, depending on the total trade volume and the matching process. The same process will then repeat for the next food commodity until all food categories have been traded. For simplicity, we assume that the trading outcome of one food category does not affect that of the other. Figure 1 illustrates the ranking and matching process of trade among countries.

One important factor in the model is trade saturation, which is the rate between realized and maximum trade amounts. Trade saturation ranges from 0% to 100%, which we vary in the computer experiments. When trade saturation is 100%, all trade opportunities are realized. Countries will keep trading until either there are no more importers with remaining demand or exporters with remaining supply. There will be no missed trading opportunities, which can be regarded as an ideal case. When trade saturation is less than 100%, not all potential trade opportunities are realized. Countries at the bottom of the trade priority may not be able to trade, even though there are outstanding buyers and sellers of the same commodity. This ‘less than ideal’ case may be closer to reality, as factors such as to risk avoidance, market failure, lack of time (for perishable food), lack of information, communication or facilitators can prevent potential trade opportunities from being realized. The higher the trade saturation, the higher percentage of trade opportunities are realized. Later we examine the impact of trade saturation on food and nutrient security across countries.

### 2.4. Design concepts

#### 2.4.1. Interaction

The countries interact with each other via trade. Links and connections are created between countries when one offers to buy from or sell to another. A country's decision to trade with another does not only affect the two countries involved in the trade, but it also indirectly affects other countries because once the food commodity has been traded, there will be less available on the market. Countries also interact with each other via their rankings of each other on trade priority. Their past interactions (trade) with other countries will influence the ranking as familiarity increases, which will in turn affect the way they interact/trade with others in the future.

#### 2.4.2. Emergence

Trade relationships can emerge through repeated interactions among countries. Countries who have never traded before (and thus are low on each other's trade priority) can start a trade relationship by chance, for example, due to the lack of available trade partners at the time. The trade experience will then encourage the countries to trade more with each other in the future, thus the emergence of new trade relationships. The nutrients available per capita in each country emerge from the trade.

#### 2.4.3. Stochasticity

The main source of randomness in the model comes from the matching of trade partners: the sequence of the countries matters when they send each other a buying offer. The random sequence of agents to execute functions is internal in NetLogo. To account for that internal stochasticity, we run the model 30 times for each parameter combination and look at the variance in results. As we can see in tables 15 to 21 in the Appendix, the variances caused by the internal stochasticity are very small. Apart from that, the model is data-driven and has not drawn other random parameters from distributions.

### 2.5. Initialization and data

The initialization of the model is based on empirical data or estimates from existing research. The 165 countries are created with initial GDP, population, production, food consumption using the average value of the years 2000, 2001 and 2002 in FAO's FBS data to smooth out any anomalies in any particular year. The past trade relationship is derived from FAO's trade data of all commodities. The geographic location and area of countries are initialized using a GIS world map. The regions and sub-regions to which countries belong are according to UN categorization and consistent with Müller *et al*. [44]. The typical diet for each country is initialized as the food consumption in 2000 from FBS. The income categories and the corresponding fortification type is derived from The World Bank and Food Fortification Initiative, respectively. The avoidable waste rate is initialized using estimates from estimation in Gustavsson *et al*. [52].

### 2.6. Input data

The current model runs from 2000 to 2013. Each year is input from data from FAO to update the GDP, population and production for all countries. The FAO FBS data is available until 2013. Table 4 shows the input data for model running from 2000 to 2013. For scenario analysis in the future, the model will use projected data for production, GDP and population between 2014 and 2050.

**Table 4.** Input data for model running from 2000 to 2013.

variable	data source	years
GDP	UN GDP of countries	2000–2013
population	UN GDP of countries	2000–2013
production (multivariate)	FAO food balance sheet: production	2000–2013

## 3. Model calibration

### 3.1. Calibration

The parameters to be calibrated are the weights given to each of the four elements (GDP per capita, distance, historic and emergent trade relationships) in the countries' evaluation of trade partner priorities, which cannot be observed in empirical data. Because the ranking of countries is relative, we lose one degree of freedom, and hence we fix the weight for distance at 1 and allow the other three parameters to vary. The sampling of the parameters is twofold: first, we draw a sample of 10 000 random parameters from a three-dimensional Latin hypercube sampling ranging between 0 and 1; second, we transform the random sample using an exponential transformation to account for the ratio relationships (i.e. relative importance) between the weights, so that the parameters range from 0.05 to 20. In other words, relative to distance (which has a fixed weight 1), the weight for the other parameters range from 0.05 (1/20 as important) to 20 (20 times as important).

We then run the model on the 10 000 sampled parameter combinations. The empirical data we use for calibration and validation is FAO FBS on import and export volume and food consumption, and trade data from the United Nation Comtrade Database.^3^ The data for validation and calibration is available between 2001 and 2013 (the model is initialized in 2000), of which the first seven years (2001–2007) is used for calibration, and the latter six years (2008–2013) for validation.

The evaluation of the model results is based on two dimensions: trade volume and trade partners. Trade volume compares the actual import, export and food consumption (in FAO FBS) in each country with the simulated results from the model. Because the simulated figures will almost surely not be the same as the observed ones (factors unaccounted for in the model, factors unobserved, errors in model specification, errors in input data, errors in empirical/validation data), we use a threshold to determine ‘match’: if the simulated volume is within ±20% of the actual volume (of import, export and food in each country), it is regarded as a match. The first dimension, trade volume, measures the percentage of simulated trade volumes (import, export, food) that falls within the range of the actual volume between 2001 and 2007.

The second dimension, trade partners, compares the actual bilateral trade between two countries in each food category with the simulated ones. If for a specific food category, the importer and exporter in the empirical data match that in the simulated data (regardless of trade volume), it is marked as a match. The trade partner index is the percentage of simulated trade records that matches the actual one of all trade records between 2001 and 2007. Because the FAO trade data does not contain information on bilateral trade partners or is not commodity-specific, we use the Comtrade data from the United Nations to calibrate and validate the bilateral trade results. The Comtrade data, however, does not cover all food categories in FAO. Of the 91 food categories, 42 are available in the Comtrade data.

**Figure 2.**
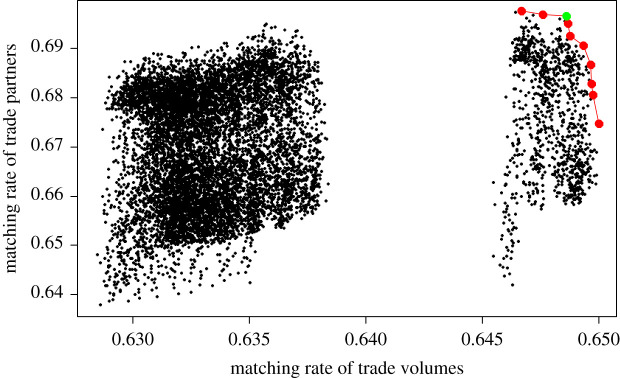
The Pareto front of the 10 000 parameter combinations.

To account for internal stochasticity, we run the model 30 times for each parameter combination and calculate the average matching rate. Figure 2 shows the 10 000 parameter combinations (consisting of three weights) plotted by the two evaluation dimensions: average matching rate for trade volume and trade partners. The higher the matching rate in each dimension, the better the model with that parameter combination performs. Tables 13 and 14 in Appendix show a small subset or example of the simulated trade volume and partners from the calibrated model (versus actual data), which we use to calculate the matching rate. The full simulation results for the calibrated model are available in the electronic supplementary material, due to the large size of the data.

Figure 2 shows the Pareto front (the red line connecting the points) of the parameter combinations. The Pareto front represents the set of optimal parameters in that the model performance cannot be improved on one dimension without lowering the performance on the other dimension. There are 10 points on the Pareto front, as shown (in red and green) in figure 2. There is a big gap in the graph, which shows that a group of parameter combinations (those to the right of the gap) does significantly better in matching trade volumes than the rest. The data used to generate the graph, i.e. the mean match rate of trade partners and trade volumes for all 10 000 parameter combinations is available in the electronic supplementary material, data.

By definition, all the points on the Pareto front are incomparable and can only be partially ordered. Since we value the two performance dimensions equally, we would like to choose a parameter combination that does relatively well in both dimensions. We then calculate the average of the two dimensions (i.e. give equal weight to each) and pick the point with the highest average (in green in figure 2), which will be the parameters for the calibrated ABM. The calibrated values of the weight for GDP, distance (fixed at 1 as the bench market), historic and emergent trade (in trade priority evaluation) are listed in table 5. We see that GDP and Historic trade have higher weights than distance and emergent trade, indicating a relatively bigger influence in countries' ranking of trade partners. However, the other two elements are also significantly different from zero, which means they also play a role. There are two caveats concerning the validation method. First, the parameters are the same for all countries (lack of parametrization per country). Second, there are potential although not linear correlations between the four elements in the ranking of trade partners as discussed in §2.2.2. However, they are not an issue for the study, which includes the prediction trade volumes among countries based on their trade relationships.

**Table 5.** Calibrated weights for GDP, distance, historic and emergent trade in trade priority evaluation.

GDP	distance (fixed)	historic trade	emergent trade
1.40	1	1.40	0.72

### 3.2. Discussion on calibration and validation

To save space, the validation results are presented in appendix A.1. Overall, we find that the model has a better predictive power for trade partners than trade volumes, which is unsurprising partly because the calibrated parameters are in the functions of countries to rank and match trade partners. Moreover, the errors in input data for the volume of trade are likely to be larger than that for trade partners. As a result, we expect the validation results for volume to be less accurate than that for trade partners. We also find that the predictive power of the model decreases over time from 2008 to 2013. As the model moves away from the original year it is initialized (2000), and the years on which is it calibrated (2001–2007), we expect its predictive power to go down, and the variances among parallel models to go up as the effects of stochasticity and random events from previous periods accumulate over time (i.e. being path-dependent).

While using empirical data to initialize, input, calibrate and validate the global trade model, we notice that the data availability varies greatly across countries, and it is the dataset available to all countries (the common denominator) that determines the data the model can use for input and validation. While some countries (e.g. UK, US) have more detailed or accurate data for trade and nutritional intake, the same data is not available for all countries, especially some countries in Africa, which largely restricts the data available for the global ABM. In some models, the problem is mitigated by grouping countries into regions and sub-regions, so missing data for some countries do not necessarily cause issues in the aggregated models. In ABM, however, because individual countries are modelled on their own, there is a higher demand for the same type of data with the same content, quality and format to be available for all countries. We identify the issue of data inequality across countries as one of the challenges in building an empirically grounded global ABM.

Finally, we find that a Pareto front can be a useful visual tool to show and compare the performance of models when the evaluation criteria are multi-dimensional. Not only can it identify a set of models with the ‘best’ performance (partially ordered), it can also reveal patterns of model performance in the evaluation space, especially when the number of candidate models is large. In this study, we select a model on the Pareto front by giving equal weights to the two evaluation criteria and pick the one with the highest weighted average. In the future, more sophisticated methods can be developed to select a model in the Pareto set; or one can include all models in the Pareto set and develop predictions based on multiple Pareto models, based on which new approaches can be developed towards model optimization, selection and prediction, which we leave for future research.

## 4. Preliminary results

### 4.1. The impact of trade on macro and micronutrient sufficiency in 2015

In this section, we show preliminary results on the impact of trade (saturation) on micro and macronutrient sufficiency. For each nutrient, we will compare the nutrition security of countries when trade saturation is low (60%, i.e. 60% of all potential trade opportunities are realized), medium (80%, i.e. 80% of all potential trade opportunities are realized) and high (100%, i.e. all potential trade opportunities are realized). Trade saturation is an exogenous intervention parameter that we will vary in the experiment. In reality, trade saturation can be influenced by countries' trade policies, trade conflicts and other factors such as a global pandemic. We will vary the level of trade saturation by low, medium and high in the computer experiment and study its impact on trade and food security.

As we discussed before in §2.2.1, the population-level nutrient intake (average per person) is calculated based on food consumption after production and trade, which we compare with nutrient requirement (average per person), which is based on the demographic compositions (age and sex) and varies across countries. If a country's average nutrient intake per person is higher than its average nutrient requirement per person, the country is considered secure in the specific nutrient, and vice versa.

For all graphs in this section except for ‘fat’, countries that are nutrient secure are in blue, and countries that are nutrient insecure are in red. Grading with the colours illustrates how far the country is from achieving nutrition security. The darker the shade of blue, the more secure the country is in that nutrient (intake ≫ maximum recommendation) and the darker the shade of red, the more insecure the country is in that nutrient (intake ≪ lower recommendation). For the nutrient ‘fat’, because the recommendation is within a range, there is a lower and upper limit (too little and too much fat can be can have negative health consequences), we use three colour schemes to show fat: red for below the lower recommendation, green for healthy, and yellow for over-consumption. In this section, we show the nutrient sufficiency in 2015 under low, medium and high trade saturations for calories, fat, vitamin A, iron and zinc, the latter three are important micronutrients, and in the Appendix, we will show that for folate, niacin, riboflavin, thiamine and vitamin C (figures 8–12).

**Figure 3.**
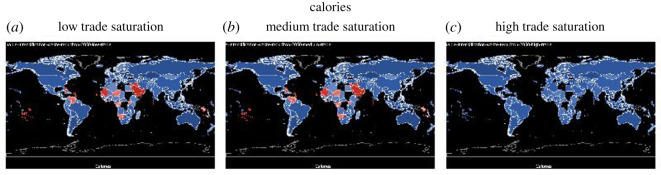
Calories sufficiency under low, medium and high trade saturation (blue = sufficient, red = insufficient).

Figure 3 shows the impact of trade on the consumption of calories. We see that with low to medium trade saturation, a handful of countries in Africa, Asia and South America will suffer from the lack of calories (or energy). The problem, however, can be solved by increasing trade saturation to 100%, meaning that the world can produce food to feed the population with enough calories, as long as all trade opportunities are realized.

**Figure 4.**
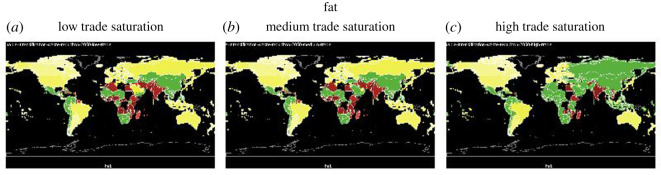
Fat sufficiency and over-consumption under low, medium and high trade saturation (green = healthy, red = insufficient, yellow = over-consumption).

Figure 4 shows the impact of trade on the consumption of fat. We see that sufficient trade not only increases fat intake to within the healthy range in some countries in Africa, Asia and South America, and it also brings the fat intake down for some countries (e.g. Russia, Southeast Asian countries) and makes them healthier. The reasons that countries reduce fat intake when the trade is fully realized is that trade allows them to access a more diverse diet from a global supply.

**Figure 5.**
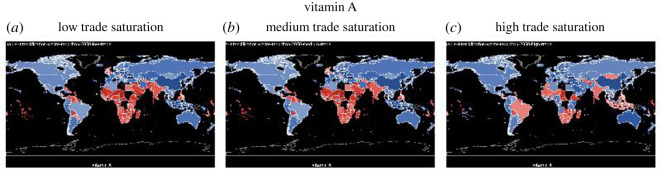
Vitamin A sufficiency under low, medium and high trade saturation (blue = sufficient, red = insufficient).

Figure 5 shows the impact of trade saturation on vitamin A supply. We see more countries including developed ones such as the UK becoming deficient in vitamin A, an essential micronutrient, under low to medium trade saturation. Increasing trade saturation reduces the level of vitamin A deficiency to a large extent, especially in African countries. It does not, however, eliminate vitamin A deficiency altogether. Moreover, some countries that are not vitamin A deficient under low and medium trade saturation such as Brazil and Indonesia become so under full trade saturation, because some vitamin A-rich foods consumed domestically under low and medium trade can now be exported when trade saturation increases. Hence for vitamin A, although trade reduces the extent of deficiency in many countries, it alone cannot eliminate deficiency.

**Figure 6.**
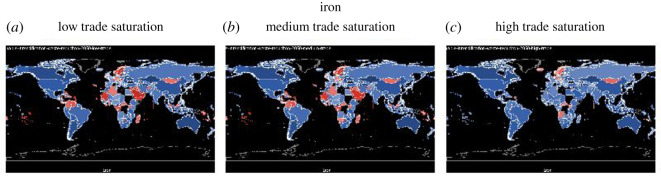
Iron sufficiency under low, medium and high trade saturation (blue = sufficient, red = insufficient).

Figure 6 shows the impact of trade saturation on iron deficiency. As with vitamin A, more countries become deficient for iron than for calories, including some Scandinavian countries under the different levels of trade. The result shows that increasing trade saturation significantly reduces the level of iron deficiency, especially in countries in Africa, Asia and South America. However, trade does not eliminate iron deficiency in Scandinavia countries or Mongolia, although it makes them less deficient (light shade in red). As with vitamin A, other measures such as improving diet and increasing production and consumption of certain foods will be needed to eliminate iron deficiency.

**Figure 7.**
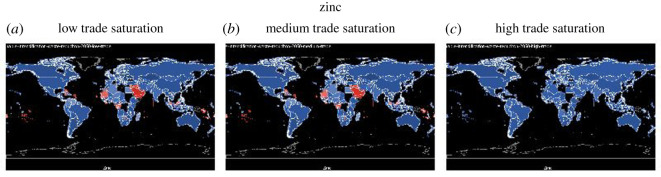
Zinc sufficiency under low, medium and high trade saturation (blue = sufficient, red = insufficient).

**Figure 8.**
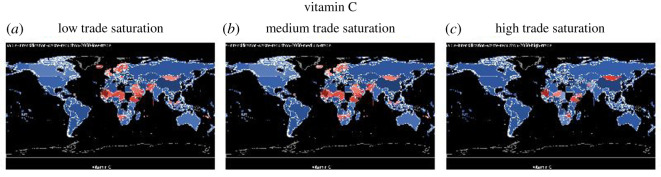
Vitamin C sufficiency under low, medium and high trade saturation (blue = sufficient, red = insufficient).

**Figure 9.**
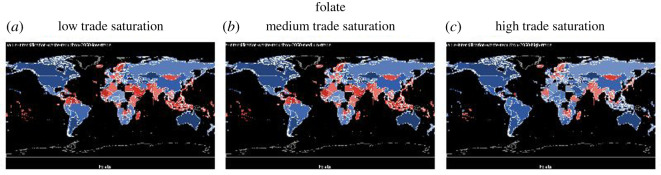
Folate sufficiency under low, medium and high trade saturation (blue = sufficient, red = insufficient).

**Figure 10.**
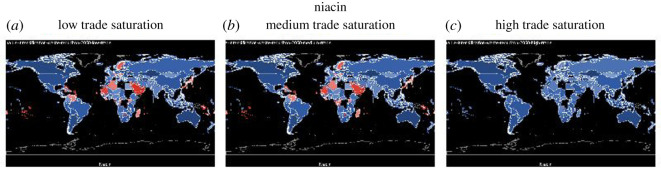
Niacin sufficiency under low, medium and high trade saturation (blue = sufficient, red = insufficient).

**Figure 11.**
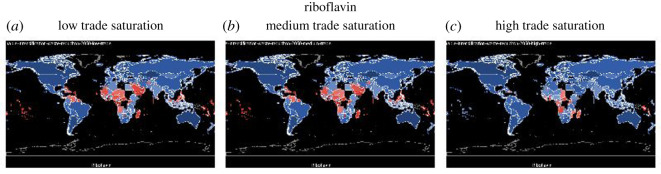
Riboflavin sufficiency under low, medium and high trade saturation (blue = sufficient, red = insufficient).

**Figure 12:**
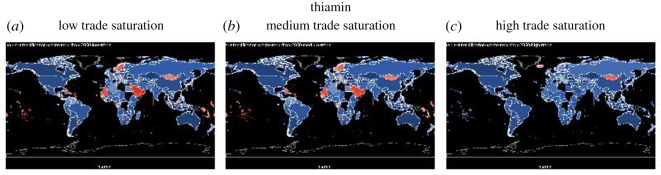
Thiamine sufficiency under low, medium and high trade saturation (blue = sufficient, red = insufficient).

Figure 7 shows the impact of trade saturation on zinc deficiency. It shows that increasing trade saturation from low or medium to high can eliminate zinc deficiency.

The results show that deficiency in micronutrients such as vitamin A and iron is much more common than that in macronutrients such as calories and fat. The latter is found only in developing countries in Africa, Asia and South America while the former can be found in both developed and developing countries, which signifies the importance of including both macro and micronutrients in the study of food and nutrition security.

We see that trade plays a positive role in improving food and nutrition security in almost all cases. A fully realized trade eliminates calories deficiency in all countries, compared with low trade and makes fat intakes more balanced across countries. For some micronutrients (e.g. niacin and zinc, see Appendix), high trade saturation has successfully eliminated deficiency in all countries, compared with low and medium trade saturation; for others, deficiency still exists even under fully realized trade, but to a lesser degree. It will require other measures, such as change increasing production and alternative trade scenarios of food rich in the micronutrients to eliminate nutrient deficiency in those micronutrients. In many developing countries currently use supplementation to correct the deficiencies. In almost all cases, we observe that trade at least reduces the level of nutrient deficiency across countries, if not eliminates it. The effect of trade is most prominent in countries most vulnerable to food and nutrition insecurity, including those in Africa, Asia and South America.

Finally, table 6 lists the nutrition intake per capita of the 20 most populous countries in 2013, which together account for more than two-thirds of the world population; also table 22 in the Appendix for the full list of countries. Note that the nutrient requirement per capita will vary slightly by countries due to the different population composition in each country (e.g. a younger/higher male percentage population will require more nutrient).

**Table 6.** Nutrition intake per capita^a^ of the 20 most populous countries in 2013, which together account for more than two-thirds of the world population (also table 22 in the Appendix for the full list of countries).

country	pop, millions	calories	protein	fat	vitamin C	vitamin A	folate	iron	zinc	thiamine	riboflavin	niacin	saturated FA
China	1384	2750	89.9	85.7	275.4	1686.9	575.9	26.3	12.3	2.60	2.06	23.77	25.32
India	1296	2543	62.4	55.3	91.4	568.3	392.8	17.0	8.3	1.63	1.27	14.23	19.73
United States	329	3869	105.1	188.6	103.1	741.6	752.1	20.0	14.3	2.64	3.38	32.18	63.68
Indonesia	262	2967	65.7	63.8	84.5	546.2	410.8	22.9	10.4	2.83	1.33	26.53	28.92
Brazil	208	3503	99.7	131.6	95.7	572.2	790.5	22.3	13.2	2.75	2.53	29.57	48.94
Pakistan	207	2738	72.4	82.5	39.6	794.9	258.8	10.4	8.1	0.94	1.66	10.24	31.98
Nigeria	203	3158	72.2	64.9	181.8	674.6	703.5	23.0	11.4	2.90	1.37	25.53	20.84
Bangladesh	159	2387	53.4	29.0	40.1	331.9	187.9	19.3	8.5	2.25	0.86	19.34	9.31
Russia	142	3347	93.7	117.0	102.5	810.5	314.4	13.5	10.7	1.39	2.04	18.99	36.70
Japan	126	2525	75.3	93.6	89.9	712.5	314.1	17.3	10.1	1.61	1.81	17.67	26.39
Mexico	125	3295	93.4	103.6	102.5	787.1	618.1	21.9	14.4	2.76	2.46	29.24	34.04
Ethiopia	108	2108	66.6	33.3	37.8	443.7	417.1	18.4	11.8	1.92	1.15	18.13	9.48
Philippines	105	2710	63.2	56.0	108.5	486.7	375.3	19.9	9.1	2.63	1.42	25.07	24.68
Egypt	99	3247	91.2	57.2	127.7	712.6	386.6	20.4	11.4	1.86	1.70	20.99	16.86
Vietnam	97	2823	80.1	71.3	155.0	885.4	451.3	27.4	11.7	3.11	1.68	27.14	24.26
Congo	85	2306	48.2	46.8	163.7	583.2	583.1	12.9	6.4	2.08	0.95	20.89	14.38
Iran	83	3478	98.9	82.0	217.3	997.7	553.7	20.9	10.8	1.83	1.87	19.67	21.87
Turkey	81	3956	111.6	129.1	174.2	1177.6	582.0	20.5	13.1	1.75	2.65	19.23	38.42
Germany	80	3787	94.8	180.8	100.3	998.6	298.8	12.8	12.0	1.65	2.87	19.32	63.85
Thailand	68	2786	60.6	57.7	120.2	624.9	244.6	21.5	8.8	1.95	1.20	19.57	21.43

^a^nutrition intake per capita will be compared with the nutrition required per capita in the country, which depends on the country's demographic composition to determine a country's food and nutrition security.

### 4.2. Discussion

The preliminary results from the model demonstrate the impact of increasing trade on improving macro and micronutrient sufficiency across countries in the world. For all nutrients included in §4.1, fully realized global trade can help ensure a sufficient supply of nutrients and enables countries to have a healthier diet. The reason is quite straightforward: trade allows the countries to access a larger variety of food, thus enabling them to have a more nutritionally balanced diet. Trade is even more important when considering micronutrient sufficiency because they tend to be more concentrated in specific foods than macronutrients such as calories and fat, so the diet needs to contain more diverse food items to supply sufficient micronutrients. The role of increasing trade in improving nutrition security is found to be universal across all macro and micronutrients, but more pronounced in micronutrients. Results from this study agree with previous studies in that global trade can balance food supply and demand across regions and smooth out nutrient intake across countries [60]. It also confirms the previous conclusion that the focus of food security should shift from calories to critical micronutrients, in which many regions will continue to have inadequacies [41].

A criticism of global food trade is that free trade penalizes the poorest and most vulnerable countries [61,62]. This is largely true: the results show that low-income countries are the first to fall into food and nutrition insecurity as trade saturation goes from high to medium to low, partly because they are often ranked lower on countries' trade priority due to their low GDP per capita and remote location. However, the results also suggest that the solution is not trade-protectionism. This is very important for countries where diets need to diversity to achieve nutrition security. We need to further remove any trade barriers and increase trade saturation so that all potential trade opportunities can be used and the demand for the most vulnerable countries can be met. Preliminary results show that increasing trade saturation always improves the nutrition security of countries. For some nutrients, trade alone can eliminate food and nutrition insecurity for all countries altogether. The poor and vulnerable countries also benefit from trade. Because they are lower on the priority rank, they benefit the most from additional trade liberalization and opportunities. Pradhan *et al*. [63] estimate that in 2000 about 1 billion people from Asia and Africa require cross-continental agricultural trade to be food secure, and by 2050 the number of people depending on trade for food security will be between 1.5 and 6 billion. Any hindrance to trade will put these people at high risk of food insecurity.

The model presented in the study is the first step towards a holistic approach to the grand challenge of food and nutrition security under global trade and climate change. There are many ways to extend the model in the future. First, the dimension and factors of countries’ trade relationships included in the model are far from complete. Apart from GDP, distance, and historic and emergent trade relationships, many factors can affect the trade relationships between countries. For example, the trade war between the US and China in 2019 was motivated by complex economic and political reasons. The flexibility of the ABM, however, means that the model can be easily adapted to implement additional factors to increase the dimension of the trade relationship. The relation-driven trade model developed in this paper will be a framework to which these relation-driven dimensions and motivations can be added to analyse its impact on trade and food security.

Moreover, so far we have only considered the average nutrition intake in a country. We have not considered the inequality and distribution within a country. A country may have enough food for its population overall, but is still food insecure because of unequal internal allocation. This is especially important for countries with unequal developments across regions, where a minority of the population has more than adequate access to food and nutrition while the majority do not. Since the global model cannot model each country in such details due to computational constraints, one approach is to use a measure of inequality of access to food within a country as used by the FAO, such as in the FEEDME model [64,65], which uses a country-level measure of inequality (income and food access inequality) to determine the percentage of a population in each country which is undernourished. Another approach is to couple the global model with individual country models for the country of interest. For example, we can couple the individual country model of China or Brazil with the global trade model developed in this paper, which allows us to position the internal dynamics and interactions between different regions and firms under the background of global trade, without having to acquire the same level of detailed data for all countries. This is particularly useful for countries like Brazil where global food trade plays a crucial role in its domestic agri-food systems.

## 5. Concluding remarks

In an increasingly connected world, a country's ability to secure food concerns more than what is produced on the ground, but also its relationships with other countries. The issue is particularly relevant and timely given the current debates about Brexit, US–China trade war and the global COVID-19 pandemic. This paper develops an empirically grounded, relation-driven model of global trade to study the impact of trade on the food and nutrition security of countries around the world, complementing existing price-driven trade models in the literature. It makes several important contributions. First, it provides a flexible modelling framework that focuses on country-wise relationships in trade. The framework can be used to study more complex and relation-driven behaviour when trading with each other, such as trust, familiarity, history and conflicts. Second, the global trade model is linked with a comprehensive nutrition formula based on food consumption to investigate the impact of trade on food and nutrition security. As researchers have been constantly calling for a holistic and multifactorial approach to these global challenges, the model is one of the first attempts to develop an integrated framework consisting of socio-economic, geopolitical, nutrition, environmental and agri-food systems to tackle these global challenges.

Preliminary results show that global trade has a significant impact on food and nutrition security across countries. Increasing trade improves the nutritional security of almost all countries, especially countries in Africa, Asia and Latin America susceptible to food insecurity. For some nutrients such as calories, niacin and zinc, trade alone can eliminate nutrition insecurity for all countries; for others such as vitamin A, folate, riboflavin and iron, trade improves nutrition security but is insufficient to achieve food security. Other measures such as dietary changes may be needed. We also find that trade allows countries to have a healthier and more balanced diet, due to the increased variety of food enabled by trade: it decreases fat intake in countries that previously consume too much fat and vice versa. Overall, we find that the effect of trade on enhancing nutrition balance and security is universal across all macro and micronutrients and countries.

In the future, the global trade model developed in the study can be coupled with individual country models to include more detailed regional dynamics, firm interactions and inequality within countries. It offers a way to link models of individual country's agri-food system with a dynamic global trade system, which is particularly useful for countries like Brazil where global food trade plays a crucial role. Finally, the model will be used to systemically conduct scenario analysis on food security under various scenarios of climate change, dietary change and de-globalization.

## Supplementary Material

Points in the pareto front in model validation; validation result in trade partners; validation result in trade volume; simulated vs actual trade partners; simulated vs actual trade volumes

Reviewer comments

## Footnotes

1 https://www.worldbank.org/

2 US Department of Agriculture, Agricultural Research Service. National Nutrient Database for Standard Reference, Release 27, 2014. Available online: http://www.ars.usda.gov/ba/bhnrc/ndl [54].

3 https://comtrade.un.org/.

## Data accessibility

We have provided the (simulated) data used in the paper as electronic supplementary material.

## Appendix A

### A.1. Validation

The data we use for validation are the same as for calibration, except that we use the year 2001–2007 for calibration and 2008–2013 for validation. We run the model 30 times with the calibrated parameters in table 5 and calculate the mean and standard deviation (s.d.) of the match rate in trade volume and trade partners among the 30 runs. The full validation results are available in tables 15 to 21.

#### A.1.1. Trade volume

Table 7 shows the percentage of the simulated volume of trade and consumption that is within ±20% of the actual volume from data. We see that, overall, the model produces a 54.80% matching rate. It does better in predicting export volume and food consumption than predicting import volume. The variance or s.d. among model runs (from internal model stochasticity) is very small, which suggests the absence of bifurcation.

**Table 7.** Overall match for trade *volume* (within ±20% of actual volume).

	mean	s.d.
all	0.5480	0.0009
import	0.4757	0.0011
export	0.6177	0.0009
food	0.5507	0.0008

Table 8 shows the matching rate of simulated volume by aggregated food category. Note that the average match rate varies across different food categories. The model's prediction of trade and consumption volumes is better for some foods (sugar crops, spices, animal fats, aquatic products) than others (vegetables, milk). Also note that the variance or s.d. tends to be higher for foods with lower average matching rate, which suggests that those foods are more subject to stochastic factors in trade, which may explain the lower matching rate in prediction.

**Table 8.** Match rate for trade *volume* by commodity.

	mean	s.d.
cereals—excluding beer	0.4915	0.0014
starchy roots	0.6046	0.0002
sugar crops	0.7997	0.0001
sugar & sweeteners	0.5569	0.0012
pulses	0.5024	0.0015
treenuts	0.6247	0.0009
oilcrops	0.5790	0.0014
vegetable oils	0.6054	0.0010
vegetables	0.3512	0.0006
fruits—excluding wine	0.4777	0.0006
stimulants	0.4134	0.0017
spices	0.6942	0.0009
alcoholic beverages	0.4336	0.0011
meat	0.4492	0.0015
offals	0.4978	0.0016
animal fats	0.6663	0.0008
milk—excluding butter	0.3212	0.0011
eggs	0.4601	0.0019
fish; seafood	0.4580	0.0008
aquatic products; other	0.8777	0.0004

Table 9 shows the matching rate of simulated volume by region. The model can better predict trade volumes for some regions (sub-Saharan Africa, Latin America) than others (North America, Europe, Pacific OECD). The regions that the model does relatively well in predicting tend to be developing regions and countries (sub-Saharan Africa, Latin America); the regions that the model does relatively poorly are developed ones (North America, Europe and Pacific OECD). The model's assumption of increasing consumption as production increases may be more valid in developing countries than developed ones; it may not capture the most recent trend in food consumption in developed countries that diverge from a concave increasing function as projected by most conventional models. Empirical data and evidence would be needed to adjust the model for that.

**Table 9.** Match rate for trade *volume* by region.

	mean	s.d.
sub-Saharan Africa	0.6253	0.0001
centrally planned Asia	0.5473	0.0004
Europe	0.4338	0.0001
former Soviet Union	0.5285	0.0003
Latin America	0.5955	0.0002
Middle East/North Africa	0.4674	0.0001
North America	0.3260	0.0004
Pacific OECD	0.4351	0.0003
Pacific Asia	0.5563	0.0003
South Asia	0.5349	0.0002

Table 10 shows the matching rate of simulated volume by year. We can see that the matching rate steadily goes down over the years, which is as expected as we move away from the original year (2000) initialized with empirical data. The variance of results from the parallel models also increases over time, which is also as expected, because stochasticity (such as emergent trade relationship) occuring in early years may have a lasting effect on model results from that point, the accumulation of which will lead to increased variance among parallel models.

**Table 10.** Match rate for trade *volume* by year.

	mean	s.d.
year2008	0.5689	0.0009
year2009	0.5642	0.0009
year2010	0.5526	0.0009
year2011	0.5419	0.0009
year2012	0.5326	0.0010
year2013	0.5279	0.0011

#### A.1.2. Trade partners

This section shows the matching rate of trade partners between simulated and actual trade outcome (importer, exporter and food traded). Table 11 shows the overall matching rate of trade partners and matching rate by year. Overall, the model correctly predicts 67.31% of all trade partners between 2008 and 2013. Similar to trade volume, the model's predictive power decreases over time from 68.95% in 2008 to 66.90% in 2013. The decrease, however, is moderate and the model still has a relatively good predictive power in 2013.

**Table 11.** Match rate for trade *partner* by year.

	mean	s.d.
overall	0.6731	0.0005
year2008	0.6895	0.0008
year2009	0.6815	0.0009
year2010	0.6584	0.0010
year2011	0.6703	0.0011
year2012	0.6697	0.0011
year2013	0.6689	0.0008

Table 12 shows the matching rate of trade partners by aggregated food category. Because the data used for validation (from Comtrade) does not cover all the food traded in the model, only the ones available in Comtrade are presented here. Similar to trade volume, the model's ability to predict trade partners varies across food categories. For some (e.g. oil crops, stimulants and spices), the model can predict correctly the majority (greater than 85%) of the trade partners, and for others (starchy roots), the model can only predict less than half. One of the reasons could be that the production of some commodities is concentrated in a handful of countries (e.g. oil crops, stimulants and spices). It is relatively easy for the model to predict correctly trade partners for those commodities, as there are only a handful of sellers, than for commodities where buyers and sellers are more scattered (tables 13–22).

**Table 12.** Match rate for trade *partner* by commodity.

	mean	s.d.
cereals—excluding beer	0.6891	0.0006
starchy roots	0.3879	0.0020
sugar crops	0.7139	0.0006
pulses	0.5391	0.0012
treenuts	0.6651	0.0017
oilcrops	0.9020	0.0004
vegetables	0.7121	0.0038
fruits—excluding wine	0.6770	0.0011
stimulants	0.9167	0.0023
spices	0.8770	0.0086

**Table 13.** Example data of simulated versus actual trade *volumes* (see electronic supplementary material for full results).

country	element	item	year	actual	simulated
Afghanistan	import	wheat and products	2000	929	1570
Afghanistan	export	wheat and products	2000	0	0
Afghanistan	food	wheat and products	2000	2600	2681
Afghanistan	import	rice (milled equivalent)	2000	219	260
Afghanistan	export	rice (milled equivalent)	2000	0	0
Afghanistan	food	rice (milled equivalent)	2000	372	410
Afghanistan	import	barley and products	2000	5	90
Afghanistan	export	barley and products	2000	0	0
Afghanistan	food	barley and products	2000	84	95
Afghanistan	import	maize and products	2000	0	63
Afghanistan	export	maize and products	2000	0	0
Afghanistan	food	maize and products	2000	66	109
Afghanistan	import	rye and products	2000	0	0
Afghanistan	export	rye and products	2000	0	0
Afghanistan	food	rye and products	2000	0	0
Afghanistan	import	oats	2000	0	0
Afghanistan	export	oats	2000	0	0
Afghanistan	food	oats	2000	0	0
Afghanistan	import	millet and products	2000	0	0
Afghanistan	export	millet and products	2000	0	0.67
Afghanistan	food	millet and products	2000	20	19
Afghanistan	import	sorghum and products	2000	0	0
Afghanistan	export	sorghum and products	2000	0	0
Afghanistan	food	sorghum and products	2000	0	0
Afghanistan	import	cereals; other	2000	0	0
Afghanistan	export	cereals; other	2000	0	0
Afghanistan	food	cereals; other	2000	0	0
Afghanistan	import	cassava and products	2000	0	0
Afghanistan	export	cassava and products	2000	0	0
Afghanistan	food	cassava and products	2000	0	0

**Table 14.** Example data of simulated versus actual trade *partners* (see electronic supplementary material for full results).

item	year	from	to
wheat and products	2000	India	Malaysia
wheat and products	2000	India	Sri Lanka
wheat and products	2000	India	Nepal
wheat and products	2000	India	Mexico
wheat and products	2000	India	Russian Federation
wheat and products	2000	India	Ukraine
wheat and products	2000	India	Iraq
wheat and products	2000	India	Italy
wheat and products	2000	India	Ethiopia
wheat and products	2000	India	Netherlands
wheat and products	2000	India	Philippines
wheat and products	2000	India	Bangladesh
wheat and products	2000	India	Brazil
wheat and products	2000	India	Algeria
wheat and products	2000	India	Republic of Korea
wheat and products	2000	India	Nigeria
wheat and products	2000	India	China
wheat and products	2000	India	Thailand
wheat and products	2000	India	Afghanistan
wheat and products	2000	India	United Arab Emirates
wheat and products	2000	India	Israel
wheat and products	2000	India	Japan
wheat and products	2000	India	Morocco
wheat and products	2000	India	Yemen
wheat and products	2000	India	Indonesia
wheat and products	2000	India	Iran (Islamic Republic of)
wheat and products	2000	India	Egypt
wheat and products	2000	Denmark	Belgium
wheat and products	2000	Denmark	Italy
wheat and products	2000	Denmark	Netherlands
wheat and products	2000	Denmark	Republic of Korea
wheat and products	2000	Denmark	Japan
wheat and products	2000	Denmark	Luxembourg
wheat and products	2000	Denmark	Israel
wheat and products	2000	Canada	Viet Nam
wheat and products	2000	Canada	Colombia
wheat and products	2000	Canada	Chile
wheat and products	2000	Canada	Malaysia
wheat and products	2000	Canada	Jamaica
wheat and products	2000	Canada	Indonesia
wheat and products	2000	Canada	El Salvador
wheat and products	2000	Canada	Panama

**Table 15.** The matching rate of the calibrated model for all trade *volumes*, and *volumes* for import, export and food consumption.

index-run	all	import	export	food
01	0.548832	0.476725	0.618373	0.551398
02	0.547023	0.474583	0.616645	0.54984
03	0.547062	0.474551	0.616891	0.549743
04	0.548839	0.476715	0.618437	0.551366
05	0.54699	0.474551	0.616667	0.549754
06	0.546823	0.474422	0.616389	0.549658
07	0.546958	0.47454	0.616592	0.549743
08	0.548797	0.476704	0.618363	0.551324
09	0.547097	0.474604	0.616859	0.549829
10	0.547087	0.474529	0.616913	0.549818
11	0.54688	0.474487	0.616453	0.5497
12	0.548885	0.476704	0.61848	0.551473
13	0.548793	0.476651	0.618426	0.551302
14	0.548924	0.476672	0.618671	0.55143
15	0.548839	0.476683	0.618384	0.551451
16	0.54885	0.476736	0.618352	0.551462
17	0.54699	0.474551	0.616677	0.549743
18	0.54891	0.476683	0.618596	0.551451
19	0.546994	0.474476	0.616752	0.549754
20	0.548903	0.476704	0.618565	0.551441
21	0.548761	0.476619	0.618416	0.551249
22	0.548761	0.476629	0.618278	0.551377
23	0.548871	0.476608	0.618628	0.551377
24	0.548868	0.476683	0.618501	0.551419
25	0.54703	0.474572	0.616699	0.549818
26	0.546933	0.474433	0.616667	0.5497
27	0.548956	0.476746	0.618639	0.551483
28	0.548786	0.476651	0.618394	0.551313
29	0.548751	0.476704	0.618235	0.551313
30	0.546891	0.474454	0.616602	0.549615
mean	0.548036	0.475746	0.617685	0.550678
s.d.	0.000941	0.001092	0.000907	0.000831

**Table 16.** The matching rate of the calibrated model for trade *volumes* for different foods (part 1).

run	cereals	starchy roots	sugar crops	sugar & sweeteners	pulses	treenuts	oilcrops	vegetable oils	vegetables	fruits
01	0.4927	0.6046	0.7998	0.5582	0.5035	0.6255	0.5798	0.6061	0.3512	0.4783
02	0.4900	0.6047	0.7996	0.5552	0.5003	0.6239	0.5772	0.6045	0.3509	0.4770
03	0.4899	0.6047	0.7996	0.5560	0.5008	0.6235	0.5779	0.6041	0.3508	0.4770
04	0.4927	0.6047	0.7998	0.5577	0.5037	0.6253	0.5800	0.6060	0.3515	0.4783
05	0.4899	0.6043	0.7996	0.5555	0.5008	0.6235	0.5779	0.6044	0.3511	0.4770
06	0.4900	0.6041	0.7996	0.5550	0.5006	0.6236	0.5776	0.6039	0.3502	0.4771
07	0.4900	0.6043	0.7996	0.5558	0.5005	0.6237	0.5776	0.6044	0.3499	0.4770
08	0.4927	0.6046	0.7998	0.5582	0.5037	0.6253	0.5800	0.6061	0.3516	0.4781
09	0.4899	0.6045	0.7996	0.5555	0.5012	0.6237	0.5769	0.6048	0.3508	0.4771
10	0.4898	0.6047	0.7996	0.5560	0.5008	0.6238	0.5772	0.6047	0.3514	0.4768
11	0.4896	0.6044	0.7996	0.5558	0.5006	0.6236	0.5776	0.6039	0.3500	0.4769
12	0.4928	0.6046	0.7998	0.5578	0.5033	0.6254	0.5801	0.6066	0.3510	0.4781
13	0.4926	0.6048	0.7998	0.5577	0.5035	0.6254	0.5800	0.6060	0.3514	0.4781
14	0.4928	0.6046	0.7998	0.5581	0.5040	0.6255	0.5803	0.6061	0.3517	0.4780
15	0.4928	0.6046	0.7998	0.5575	0.5036	0.6254	0.5803	0.6063	0.3515	0.4782
16	0.4927	0.6048	0.7998	0.5578	0.5038	0.6254	0.5805	0.6063	0.3514	0.4782
17	0.4900	0.6042	0.7996	0.5558	0.5006	0.6235	0.5776	0.6042	0.3511	0.4770
18	0.4927	0.6042	0.7998	0.5581	0.5041	0.6253	0.5803	0.6064	0.3516	0.4782
19	0.4898	0.6042	0.7996	0.5555	0.5007	0.6235	0.5776	0.6044	0.3516	0.4771
20	0.4926	0.6047	0.7998	0.5577	0.5035	0.6255	0.5801	0.6066	0.3510	0.4784
21	0.4927	0.6045	0.7998	0.5578	0.5039	0.6254	0.5801	0.6057	0.3516	0.4782
22	0.4928	0.6048	0.7998	0.5577	0.5036	0.6255	0.5800	0.6060	0.3517	0.4780
23	0.4929	0.6048	0.7998	0.5579	0.5041	0.6255	0.5801	0.6059	0.3517	0.4781
24	0.4927	0.6048	0.7998	0.5572	0.5040	0.6256	0.5803	0.6062	0.3520	0.4782
25	0.4898	0.6043	0.7996	0.5557	0.5006	0.6237	0.5772	0.6045	0.3513	0.4769
26	0.4900	0.6045	0.7996	0.5553	0.5008	0.6236	0.5777	0.6041	0.3507	0.4771
27	0.4927	0.6046	0.7998	0.5583	0.5037	0.6254	0.5800	0.6065	0.3529	0.4784
28	0.4926	0.6045	0.7998	0.5578	0.5039	0.6254	0.5801	0.6060	0.3502	0.4784
29	0.4928	0.6048	0.7998	0.5580	0.5033	0.6255	0.5798	0.6060	0.3510	0.4782
30	0.4898	0.6045	0.7996	0.5553	0.5010	0.6239	0.5771	0.6040	0.3505	0.4769
mean	0.4915	0.6046	0.7997	0.5569	0.5024	0.6247	0.5790	0.6054	0.3512	0.4777
s.d.	0.0014	0.0002	0.0001	0.0012	0.0015	0.0009	0.0014	0.0010	0.0006	0.0006

**Table 17.** The matching rate of the calibrated model for trade *volumes* for different foods (part 2).

run	stimulants	spices	alcoholic beverages	meat	offals	animal fats	milk—excluding butter	eggs	fish; seafood	aquatic products
01	0.4150	0.6949	0.4346	0.4506	0.4987	0.6672	0.3222	0.4596	0.4587	0.8778
02	0.4118	0.6930	0.4328	0.4475	0.4963	0.6654	0.3201	0.4570	0.4571	0.8771
03	0.4116	0.6933	0.4331	0.4474	0.4963	0.6657	0.3201	0.4614	0.4572	0.8775
04	0.4152	0.6950	0.4352	0.4506	0.4990	0.6668	0.3219	0.4606	0.4587	0.8782
05	0.4116	0.6933	0.4325	0.4475	0.4959	0.6654	0.3198	0.4570	0.4573	0.8773
06	0.4115	0.6930	0.4322	0.4476	0.4959	0.6654	0.3201	0.4570	0.4570	0.8771
07	0.4112	0.6931	0.4319	0.4474	0.4963	0.6653	0.3198	0.4597	0.4572	0.8774
08	0.4150	0.6951	0.4343	0.4500	0.4990	0.6669	0.3222	0.4613	0.4587	0.8782
09	0.4115	0.6931	0.4325	0.4478	0.4966	0.6654	0.3198	0.4593	0.4573	0.8773
10	0.4115	0.6933	0.4326	0.4478	0.4959	0.6652	0.3201	0.4587	0.4571	0.8775
11	0.4114	0.6933	0.4320	0.4474	0.4956	0.6655	0.3201	0.4610	0.4573	0.8771
12	0.4150	0.6951	0.4343	0.4507	0.4990	0.6670	0.3219	0.4616	0.4587	0.8783
13	0.4147	0.6953	0.4345	0.4506	0.4990	0.6668	0.3222	0.4616	0.4588	0.8781
14	0.4148	0.6951	0.4349	0.4506	0.4997	0.6671	0.3219	0.4633	0.4588	0.8780
15	0.4148	0.6951	0.4346	0.4507	0.4997	0.6667	0.3222	0.4616	0.4583	0.8780
16	0.4148	0.6949	0.4342	0.4506	0.4997	0.6667	0.3222	0.4606	0.4587	0.8781
17	0.4115	0.6935	0.4332	0.4472	0.4966	0.6654	0.3201	0.4587	0.4569	0.8775
18	0.4150	0.6949	0.4345	0.4508	0.4993	0.6669	0.3226	0.4620	0.4587	0.8781
19	0.4116	0.6931	0.4323	0.4476	0.4963	0.6654	0.3201	0.4583	0.4571	0.8774
20	0.4149	0.6948	0.4348	0.4503	0.4990	0.6669	0.3219	0.4640	0.4588	0.8781
21	0.4147	0.6950	0.4350	0.4504	0.4993	0.6665	0.3222	0.4616	0.4587	0.8778
22	0.4149	0.6950	0.4342	0.4500	0.4987	0.6670	0.3222	0.4606	0.4587	0.8778
23	0.4150	0.6949	0.4343	0.4510	0.4990	0.6673	0.3219	0.4609	0.4585	0.8780
24	0.4146	0.6952	0.4348	0.4504	0.4997	0.6671	0.3219	0.4593	0.4586	0.8782
25	0.4117	0.6932	0.4325	0.4480	0.4963	0.6654	0.3198	0.4587	0.4572	0.8771
26	0.4115	0.6930	0.4322	0.4474	0.4953	0.6656	0.3198	0.4580	0.4572	0.8773
27	0.4147	0.6951	0.4343	0.4505	0.4990	0.6670	0.3226	0.4616	0.4586	0.8781
28	0.4148	0.6950	0.4343	0.4506	0.4990	0.6670	0.3222	0.4626	0.4586	0.8779
29	0.4148	0.6949	0.4348	0.4502	0.4990	0.6672	0.3219	0.4582	0.4584	0.8780
30	0.4117	0.6932	0.4320	0.4472	0.4959	0.6657	0.3198	0.4577	0.4569	0.8774
mean	0.4134	0.6942	0.4336	0.4492	0.4978	0.6663	0.3212	0.4601	0.4580	0.8777
s.d.	0.0017	0.0009	0.0011	0.0015	0.0016	0.0008	0.0011	0.0019	0.0008	0.0004

**Table 18.** The matching rate of the calibrated model for trade *volumes* for different regions (AFR: sub-Saharan Africa; CPA: centrally planned Asia; EUR: Europe; FSU: Former Soviet Union; LAM: Latin America; MEA: the Middle East/North Africa; NAM: North America; PAO: Pacific OECD; PAS: Pacific Asia; SAS: South Asia).

run	AFR	CPA	EUR	FSU	LAM	MEA	NAM	PAO	PAS	SAS
01	0.6254	0.5470	0.4337	0.5282	0.5956	0.4674	0.3272	0.4353	0.5559	0.5351
02	0.6252	0.5473	0.4338	0.5284	0.5959	0.4674	0.3257	0.4351	0.5562	0.5345
03	0.6254	0.5474	0.4338	0.5284	0.5956	0.4676	0.3263	0.4355	0.5566	0.5347
04	0.6253	0.5476	0.4338	0.5286	0.5955	0.4675	0.3251	0.4349	0.5565	0.5349
05	0.6253	0.5470	0.4338	0.5282	0.5957	0.4673	0.3251	0.4353	0.5565	0.5347
06	0.6252	0.5467	0.4337	0.5279	0.5951	0.4673	0.3263	0.4351	0.5566	0.5347
07	0.6251	0.5476	0.4337	0.5287	0.5955	0.4675	0.3263	0.4349	0.5563	0.5348
08	0.6252	0.5476	0.4338	0.5283	0.5956	0.4674	0.3257	0.4357	0.5559	0.5353
09	0.6253	0.5464	0.4340	0.5287	0.5957	0.4677	0.3260	0.4351	0.5562	0.5346
10	0.6254	0.5477	0.4337	0.5283	0.5958	0.4673	0.3260	0.4349	0.5567	0.5351
11	0.6251	0.5475	0.4337	0.5288	0.5953	0.4673	0.3263	0.4349	0.5562	0.5343
12	0.6252	0.5475	0.4338	0.5287	0.5956	0.4675	0.3263	0.4353	0.5566	0.5347
13	0.6254	0.5470	0.4338	0.5280	0.5955	0.4674	0.3266	0.4347	0.5560	0.5348
14	0.6254	0.5474	0.4338	0.5290	0.5955	0.4675	0.3263	0.4353	0.5565	0.5351
15	0.6254	0.5480	0.4337	0.5284	0.5957	0.4674	0.3260	0.4349	0.5560	0.5348
16	0.6254	0.5475	0.4338	0.5284	0.5957	0.4676	0.3257	0.4353	0.5559	0.5348
17	0.6250	0.5470	0.4338	0.5290	0.5955	0.4677	0.3263	0.4355	0.5562	0.5348
18	0.6254	0.5470	0.4338	0.5286	0.5957	0.4674	0.3260	0.4347	0.5562	0.5355
19	0.6252	0.5468	0.4338	0.5289	0.5956	0.4676	0.3260	0.4349	0.5560	0.5347
20	0.6255	0.5468	0.4336	0.5286	0.5959	0.4672	0.3254	0.4351	0.5568	0.5350
21	0.6253	0.5470	0.4336	0.5287	0.5953	0.4673	0.3257	0.4347	0.5565	0.5350
22	0.6253	0.5469	0.4337	0.5287	0.5954	0.4674	0.3254	0.4351	0.5561	0.5351
23	0.6254	0.5478	0.4338	0.5287	0.5955	0.4675	0.3260	0.4345	0.5563	0.5350
24	0.6253	0.5478	0.4338	0.5288	0.5955	0.4674	0.3263	0.4353	0.5564	0.5348
25	0.6252	0.5474	0.4338	0.5282	0.5958	0.4675	0.3260	0.4349	0.5564	0.5347
26	0.6252	0.5469	0.4337	0.5284	0.5953	0.4676	0.3260	0.4353	0.5565	0.5348
27	0.6254	0.5480	0.4338	0.5283	0.5959	0.4677	0.3263	0.4357	0.5561	0.5348
28	0.6253	0.5471	0.4337	0.5289	0.5953	0.4674	0.3257	0.4357	0.5563	0.5351
29	0.6252	0.5473	0.4340	0.5283	0.5954	0.4674	0.3257	0.4355	0.5558	0.5348
30	0.6252	0.5477	0.4337	0.5280	0.5951	0.4673	0.3257	0.4355	0.5567	0.5350
mean	0.6253	0.5473	0.4338	0.5285	0.5955	0.4674	0.3260	0.4351	0.5563	0.5349
s.d.	0.0001	0.0004	0.0001	0.0003	0.0002	0.0001	0.0004	0.0003	0.0003	0.0002

**Table 19.** The matching rate of the calibrated model for trade *volumes* for different years.

run	year2008	year2009	year2010	year2011	year2012	year2013
01	0.5697	0.5649	0.5534	0.5426	0.5336	0.5289
02	0.5681	0.5633	0.5517	0.5410	0.5313	0.5267
03	0.5680	0.5635	0.5516	0.5411	0.5313	0.5269
04	0.5697	0.5650	0.5533	0.5428	0.5334	0.5288
05	0.5681	0.5631	0.5517	0.5409	0.5315	0.5267
06	0.5678	0.5631	0.5516	0.5405	0.5314	0.5265
07	0.5678	0.5633	0.5516	0.5409	0.5315	0.5266
08	0.5695	0.5650	0.5534	0.5427	0.5335	0.5287
09	0.5681	0.5633	0.5517	0.5412	0.5316	0.5267
10	0.5680	0.5634	0.5517	0.5411	0.5314	0.5270
11	0.5678	0.5632	0.5516	0.5407	0.5312	0.5268
12	0.5696	0.5650	0.5536	0.5428	0.5336	0.5288
13	0.5699	0.5647	0.5533	0.5426	0.5335	0.5288
14	0.5698	0.5652	0.5534	0.5428	0.5335	0.5290
15	0.5697	0.5649	0.5535	0.5428	0.5335	0.5287
16	0.5697	0.5649	0.5533	0.5429	0.5334	0.5289
17	0.5678	0.5632	0.5517	0.5409	0.5315	0.5267
18	0.5698	0.5651	0.5532	0.5431	0.5335	0.5288
19	0.5678	0.5632	0.5517	0.5410	0.5316	0.5267
20	0.5699	0.5650	0.5534	0.5430	0.5335	0.5287
21	0.5698	0.5649	0.5532	0.5425	0.5335	0.5287
22	0.5697	0.5651	0.5533	0.5426	0.5333	0.5287
23	0.5697	0.5652	0.5532	0.5428	0.5335	0.5287
24	0.5697	0.5650	0.5533	0.5427	0.5336	0.5290
25	0.5678	0.5631	0.5518	0.5411	0.5315	0.5269
26	0.5680	0.5633	0.5516	0.5408	0.5315	0.5264
27	0.5697	0.5652	0.5535	0.5428	0.5337	0.5288
28	0.5697	0.5649	0.5533	0.5427	0.5334	0.5287
29	0.5696	0.5649	0.5532	0.5424	0.5335	0.5289
30	0.5678	0.5635	0.5514	0.5407	0.5315	0.5265
mean	0.5689	0.5642	0.5526	0.5419	0.5326	0.5279
s.d.	0.0009	0.0009	0.0009	0.0009	0.0010	0.0011

**Table 20.** The matching rate of the calibrated model for trade *partners* for different food categories.

run	overall	cereals	starchy roots	sugar crops	pulses	treenuts	oilcrops	vegetables	fruits	stimulants	spices
01	0.6727	0.6890	0.3892	0.7141	0.5362	0.6628	0.9020	0.7090	0.6769	0.9199	0.8655
02	0.6728	0.6900	0.3877	0.7146	0.5394	0.6644	0.9023	0.7115	0.6750	0.9167	0.8739
03	0.6728	0.6883	0.3914	0.7138	0.5379	0.6636	0.9013	0.7113	0.6765	0.9187	0.8917
04	0.6728	0.6894	0.3881	0.7141	0.5407	0.6638	0.9017	0.7055	0.6763	0.9192	0.8655
05	0.6732	0.6886	0.3897	0.7143	0.5373	0.6677	0.9022	0.7083	0.6765	0.9191	0.8655
06	0.6729	0.6882	0.3871	0.7136	0.5397	0.6669	0.9014	0.7124	0.6770	0.9178	0.8750
07	0.6732	0.6908	0.3883	0.7130	0.5378	0.6638	0.9019	0.7113	0.6771	0.9153	0.8824
08	0.6733	0.6887	0.3876	0.7136	0.5394	0.6654	0.9025	0.7158	0.6770	0.9187	0.8729
09	0.6733	0.6885	0.3905	0.7148	0.5398	0.6663	0.9023	0.7080	0.6760	0.9199	0.8833
10	0.6742	0.6891	0.3903	0.7144	0.5389	0.6667	0.9026	0.7180	0.6778	0.9175	0.8833
11	0.6725	0.6888	0.3874	0.7137	0.5389	0.6648	0.9019	0.7145	0.6743	0.9177	0.8908
12	0.6726	0.6892	0.3851	0.7132	0.5382	0.6640	0.9013	0.7122	0.6777	0.9157	0.8824
13	0.6728	0.6894	0.3865	0.7138	0.5396	0.6638	0.9023	0.7134	0.6768	0.9171	0.8750
14	0.6729	0.6891	0.3865	0.7145	0.5410	0.6640	0.9019	0.7127	0.6782	0.9135	0.8908
15	0.6732	0.6886	0.3859	0.7123	0.5405	0.6652	0.9023	0.7163	0.6783	0.9165	0.8824
16	0.6727	0.6894	0.3871	0.7133	0.5397	0.6654	0.9020	0.7074	0.6783	0.9124	0.8750
17	0.6733	0.6889	0.3942	0.7145	0.5398	0.6634	0.9022	0.7097	0.6779	0.9147	0.8833
18	0.6737	0.6889	0.3872	0.7139	0.5414	0.6668	0.9019	0.7106	0.6788	0.9172	0.8824
19	0.6728	0.6891	0.3872	0.7138	0.5383	0.6648	0.9026	0.7116	0.6754	0.9206	0.8678
20	0.6728	0.6891	0.3876	0.7139	0.5384	0.6610	0.9022	0.7102	0.6780	0.9181	0.8843
21	0.6737	0.6900	0.3880	0.7142	0.5395	0.6680	0.9017	0.7097	0.6777	0.9151	0.8667
22	0.6725	0.6886	0.3857	0.7130	0.5385	0.6649	0.9020	0.7130	0.6763	0.9173	0.8655
23	0.6724	0.6889	0.3881	0.7130	0.5400	0.6649	0.9022	0.7125	0.6769	0.9106	0.8667
24	0.6732	0.6892	0.3865	0.7131	0.5386	0.6636	0.9017	0.7167	0.6783	0.9158	0.8824
25	0.6735	0.6902	0.3902	0.7140	0.5389	0.6653	0.9017	0.7093	0.6781	0.9144	0.8655
26	0.6736	0.6888	0.3892	0.7139	0.5372	0.6683	0.9019	0.7158	0.6774	0.9152	0.8655
27	0.6719	0.6884	0.3862	0.7143	0.5400	0.6636	0.9020	0.7035	0.6759	0.9172	0.8824
28	0.6732	0.6887	0.3861	0.7137	0.5388	0.6659	0.9016	0.7178	0.6766	0.9176	0.8843
29	0.6737	0.6900	0.3872	0.7146	0.5392	0.6657	0.9020	0.7168	0.6776	0.9153	0.8833
30	0.6735	0.6896	0.3862	0.7145	0.5388	0.6668	0.9022	0.7186	0.6764	0.9159	0.8739
mean	0.6731	0.6891	0.3879	0.7139	0.5391	0.6651	0.9020	0.7121	0.6770	0.9167	0.8770
s.d.	0.0005	0.0006	0.0020	0.0006	0.0012	0.0017	0.0004	0.0038	0.0011	0.0023	0.0086

**Table 21.** The matching rate of the calibrated model for trade *partners* for different years.

run	year2008	year2009	year2010	year2011	year2012	year2013
01	0.6888	0.6803	0.6578	0.6698	0.6710	0.6685
02	0.6899	0.6802	0.6587	0.6696	0.6693	0.6691
03	0.6896	0.6816	0.6572	0.6689	0.6705	0.6692
04	0.6894	0.6818	0.6576	0.6679	0.6718	0.6683
05	0.6901	0.6832	0.6592	0.6697	0.6692	0.6678
06	0.6889	0.6818	0.6587	0.6706	0.6690	0.6685
07	0.6892	0.6818	0.6584	0.6698	0.6694	0.6704
08	0.6897	0.6819	0.6596	0.6693	0.6699	0.6689
09	0.6878	0.6837	0.6577	0.6697	0.6713	0.6695
10	0.6900	0.6835	0.6592	0.6707	0.6716	0.6699
11	0.6886	0.6807	0.6586	0.6708	0.6684	0.6679
12	0.6895	0.6805	0.6581	0.6712	0.6682	0.6683
13	0.6890	0.6811	0.6578	0.6684	0.6703	0.6701
14	0.6903	0.6809	0.6570	0.6713	0.6698	0.6680
15	0.6904	0.6805	0.6592	0.6704	0.6705	0.6683
16	0.6893	0.6811	0.6567	0.6713	0.6689	0.6689
17	0.6896	0.6817	0.6601	0.6698	0.6697	0.6687
18	0.6907	0.6816	0.6595	0.6714	0.6698	0.6689
19	0.6884	0.6807	0.6593	0.6696	0.6692	0.6694
20	0.6907	0.6805	0.6593	0.6692	0.6693	0.6676
21	0.6888	0.6808	0.6594	0.6717	0.6708	0.6706
22	0.6892	0.6819	0.6567	0.6711	0.6674	0.6688
23	0.6891	0.6806	0.6578	0.6684	0.6698	0.6687
24	0.6892	0.6814	0.6581	0.6717	0.6694	0.6691
25	0.6903	0.6823	0.6593	0.6708	0.6690	0.6690
26	0.6901	0.6821	0.6586	0.6713	0.6703	0.6691
27	0.6895	0.6805	0.6571	0.6698	0.6671	0.6675
28	0.6895	0.6814	0.6589	0.6702	0.6695	0.6696
29	0.6912	0.6825	0.6573	0.6721	0.6694	0.6696
30	0.6889	0.6818	0.6591	0.6716	0.6702	0.6689
mean	0.6895	0.6815	0.6584	0.6703	0.6697	0.6689
s.d.	0.0008	0.0009	0.0010	0.0011	0.0011	0.0008

**Table 22.** Nutrition intake per capita of countries in 2013.

country	calories	protein	fat	vitamin C	vitamin A	folate	iron	zinc	thiamin	riboflavin	niacin	saturated FA
Afghanistan	2144	67	36	35	369	237	12.4	8.8	1.3	1.2	14.3	12.5
Albania	3545	112	110	190	1295	484	19.7	14.5	1.5	3.9	19.1	38.7
Algeria	3615	101	83	147	805	487	17.7	11.5	1.6	2.3	17.4	25.3
Angola	2330	52	48	159	728	390	11.4	7.7	1.6	0.7	16.4	14.6
Antigua and Barbuda	1847	71	45	138	494	578	13.8	7.5	1.8	1.7	24.8	16.9
Argentina	3253	97	119	82	769	901	21.2	12.1	2.9	3.1	32.9	38.5
Armenia	2904	89	96	244	1474	456	18.0	10.6	1.3	2.7	15.9	32.3
Australia	3413	98	171	87	1058	642	19.3	12.7	2.3	3.0	29.9	55.3
Austria	4027	98	206	130	980	335	14.2	12.3	1.8	2.9	20.4	69.5
Azerbaijan	3023	93	57	125	799	360	13.9	10.0	1.2	2.0	15.1	21.6
Bahamas	2705	84	106	251	868	560	20.1	11.2	2.3	2.3	28.1	38.5
Bangladesh	2387	53	29	40	332	188	19.3	8.5	2.3	0.9	19.3	9.3
Barbados	2975	88	92	111	702	768	19.2	9.9	2.5	2.4	30.6	33.3
Belarus	3308	91	140	136	988	349	17.3	12.4	1.9	2.1	22.6	41.0
Belgium	3976	94	191	118	1973	341	14.1	11.3	1.5	2.7	17.9	74.3
Belize	2774	76	81	144	386	728	19.0	10.0	2.8	2.0	24.7	24.6
Benin	2917	70	61	182	680	516	20.5	11.5	2.7	1.0	26.2	14.8
Bermuda	2148	83	67	237	912	323	13.1	9.4	1.3	1.9	19.0	24.7
Bolivia (Plurinational State of)	2559	75	73	75	369	591	18.9	10.5	2.5	1.8	27.0	21.8
Bosnia and Herzegovina	3129	94	80	197	1411	502	22.9	13.5	2.1	2.8	22.4	25.2
Botswana	2381	64	66	84	514	293	15.2	9.5	1.3	1.5	15.6	17.7
Brazil	3503	100	132	96	572	791	22.3	13.2	2.7	2.5	29.6	48.9
Brunei Darussalam	3025	94	93	114	909	278	19.6	12.4	1.8	2.0	26.5	31.9
Bulgaria	2907	79	108	76	603	262	11.6	9.1	1.3	2.0	15.9	32.4
Burkina Faso	3383	91	71	23	367	596	25.0	14.6	2.9	1.6	32.0	16.4
Cabo Verde	2565	73	68	120	794	667	20.1	10.1	2.6	2.1	24.4	23.7
Cambodia	2543	60	34	57	338	248	20.5	9.3	2.4	1.0	21.1	10.5
Cameroon	3016	77	66	159	807	827	24.6	12.0	2.8	1.3	27.3	16.7
Canada	3728	99	177	121	839	882	22.3	13.3	2.9	3.1	31.2	54.4
Central African Republic	2116	46	65	125	323	358	11.9	8.4	1.7	0.7	14.5	15.0
Chad	2328	72	54	32	185	428	19.5	12.5	2.1	1.1	23.5	10.7
Chile	3228	94	99	85	506	980	22.9	11.3	3.4	2.8	34.3	33.6
China; mainland	2750	90	86	275	1687	576	26.3	12.3	2.6	2.1	23.8	25.3
Colombia	2984	68	90	127	768	487	15.9	9.2	1.9	1.8	21.6	33.2
Congo	2306	48	47	164	583	583	12.9	6.4	2.1	0.9	20.9	14.4
Costa Rica	3119	78	108	116	808	601	17.6	10.1	2.2	2.4	22.4	40.5
Croatia	3234	85	137	85	727	256	11.9	10.3	1.4	2.7	15.9	47.1
Cuba	3505	93	75	183	915	979	28.0	12.5	3.4	2.4	29.2	26.3
Cyprus	2576	73	113	90	580	254	11.3	8.8	1.3	1.8	15.6	31.3
Czechia	3442	84	159	81	847	282	11.8	10.1	1.4	2.2	17.5	49.3
Democratic People's Republic of Korea	2213	60	38	128	683	411	21.2	10.0	2.3	1.1	18.8	9.3
Denmark	3733	106	154	123	1248	342	16.8	14.2	1.6	3.2	21.0	59.3
Djibouti	2585	65	55	67	687	1022	22.1	7.4	3.2	2.3	27.8	18.7
Dominica	2636	70	51	336	805	805	18.1	7.9	2.6	2.0	25.9	23.2
Dominican Republic	2750	61	95	167	780	546	16.4	7.8	1.8	1.4	20.8	37.3
Ecuador	2483	64	103	63	784	441	13.9	8.2	1.9	1.8	20.3	38.7
Egypt	3247	91	57	128	713	387	20.4	11.4	1.9	1.7	21.0	16.9
El Salvador	2662	74	70	74	471	604	18.6	10.9	2.2	2.0	21.5	25.4
Estonia	3162	98	101	118	956	382	16.3	13.0	1.7	2.9	19.7	36.7
Ethiopia	2108	67	33	38	444	417	18.4	11.8	1.9	1.2	18.1	9.5
Fiji	3183	76	103	89	507	892	22.6	9.3	3.0	1.8	29.9	54.3
Finland	3343	109	144	103	1075	750	18.2	13.5	2.7	4.4	29.7	56.5
France	3628	102	168	127	1137	318	15.1	12.8	1.5	2.6	19.2	60.4
French Polynesia	2992	94	123	109	667	283	14.8	11.3	1.4	1.7	23.9	49.8
Gabon	2780	80	62	140	640	343	14.8	9.2	1.7	1.0	24.4	17.1
Gambia	2818	65	74	22	502	270	16.1	9.6	1.9	1.2	21.8	19.5
Georgia	3057	86	72	67	470	251	12.1	9.7	1.3	1.9	15.1	23.3
Germany	3787	95	181	100	999	299	12.8	12.0	1.6	2.9	19.3	63.8
Ghana	3179	64	52	272	676	641	20.7	10.5	2.6	0.9	25.0	17.1
Greece	3543	103	161	170	1039	422	17.4	12.8	1.6	2.9	19.0	44.2
Grenada	2149	68	49	131	425	665	15.2	7.1	1.9	1.8	23.7	21.6
Guatemala	2511	65	61	72	434	592	18.0	10.2	2.3	1.7	20.8	18.2
Guinea	2656	53	62	134	709	401	18.0	8.9	2.3	1.0	20.7	18.1
Guinea-Bissau	2364	46	65	56	395	216	15.3	7.4	1.9	0.8	16.6	22.0
Guyana	3039	86	70	98	588	738	24.8	11.2	2.8	2.4	30.2	35.8
Haiti	2010	46	49	83	677	474	14.0	7.2	1.8	0.8	14.6	15.2
Honduras	2550	62	72	67	733	297	12.8	9.4	1.5	1.4	15.7	25.1
Hungary	3197	77	156	81	876	255	11.0	8.9	1.2	1.9	14.9	51.2
Iceland	2858	100	113	102	960	247	11.6	11.8	1.2	2.7	21.2	48.4
India	2543	62	55	91	568	393	17.0	8.3	1.6	1.3	14.2	19.7
Indonesia	2967	66	64	84	546	411	22.9	10.4	2.8	1.3	26.5	28.9
Iran (Islamic Republic of)	3478	99	82	217	998	554	20.9	10.8	1.8	1.9	19.7	21.9
Iraq	2546	65	60	85	631	1120	23.6	6.8	3.4	2.4	30.9	15.3
Ireland	3648	104	144	152	964	368	13.6	12.7	1.7	2.8	21.2	54.8
Israel	3775	121	164	159	1186	502	20.8	15.0	1.7	2.8	25.9	48.3
Italy	4010	109	189	146	1113	393	15.7	12.7	1.7	3.0	18.7	60.9
Jamaica	2796	77	83	159	1022	769	21.7	8.8	2.6	2.2	29.7	37.1
Japan	2525	75	94	90	712	314	17.3	10.1	1.6	1.8	17.7	26.4
Jordan	2740	70	87	84	763	319	12.1	7.5	1.1	1.3	13.6	24.5
Kazakhstan	3217	96	132	159	1152	927	23.7	12.4	2.9	3.5	30.1	38.5
Kenya	2423	66	52	86	756	645	19.8	10.7	2.4	1.8	21.4	18.4
Kiribati	2826	60	102	93	344	577	20.5	7.3	2.3	1.1	24.4	77.1
Kuwait	3310	102	112	173	1211	1029	29.2	12.9	3.2	3.2	35.9	36.2
Kyrgyzstan	2870	87	67	104	777	367	14.5	10.9	1.4	2.1	14.9	24.0
Lao People's Democratic Republic	2747	67	37	208	1111	430	28.0	11.1	3.0	1.8	26.5	9.2
Latvia	3277	88	140	107	1081	309	15.3	11.7	1.7	2.4	20.7	49.5
Lebanon	2935	79	99	147	695	416	15.3	9.8	1.2	1.9	15.2	25.6
Lesotho	2789	80	40	46	228	285	20.1	14.8	2.5	1.4	25.5	9.5
Liberia	2274	38	60	92	786	248	13.0	6.1	1.8	0.6	15.9	22.5
Lithuania	3643	111	127	98	995	351	13.7	12.7	1.8	3.1	23.3	48.6
Luxembourg	3383	103	123	174	954	341	16.4	14.3	1.7	3.5	23.1	45.1
Madagascar	2080	42	23	102	378	222	13.5	7.4	1.9	0.7	15.8	8.6
Malawi	2628	70	46	99	220	493	19.8	13.3	2.7	1.2	24.9	9.9
Malaysia	3109	85	94	76	943	251	19.1	10.2	1.8	1.4	23.6	38.1
Maldives	2369	92	56	122	615	262	14.6	8.7	1.5	1.9	24.3	18.9
Mali	2901	87	64	61	601	582	24.4	14.2	2.8	1.9	27.6	18.3
Malta	3914	118	129	173	1044	476	18.7	13.9	1.9	2.8	22.9	42.4
Mauritania	3026	85	73	27	659	338	14.2	10.2	1.4	1.7	15.3	27.8
Mauritius	3217	90	101	77	541	345	15.1	9.7	1.4	1.6	19.2	31.6
Mexico	3295	93	104	103	787	618	21.9	14.4	2.8	2.5	29.2	34.0
Mongolia	2442	86	75	59	709	239	12.4	11.0	1.0	1.8	16.0	27.9
Morocco	3634	103	75	111	626	1503	33.4	12.4	4.8	3.5	45.6	20.9
Mozambique	2515	50	45	134	433	488	14.1	9.2	2.3	0.8	18.4	12.8
Myanmar	2746	78	71	86	587	377	21.2	10.5	2.4	1.3	24.2	21.0
Namibia	2375	62	54	87	358	399	15.3	8.6	1.6	1.2	17.3	14.6
Nepal	2896	76	59	144	723	694	30.1	12.6	3.3	1.9	28.5	16.5
Netherlands	3325	99	144	144	1031	310	12.4	12.0	1.6	2.9	18.9	52.6
New Caledonia	2777	80	117	119	795	242	11.5	9.2	1.2	1.7	18.3	44.4
New Zealand	3157	88	131	114	1004	325	14.5	11.3	1.4	2.0	20.3	48.0
Nicaragua	2746	71	66	33	400	590	18.2	10.8	2.4	1.7	22.1	21.7
Niger	2999	102	65	59	481	949	27.6	15.7	3.0	2.1	32.1	17.1
Nigeria	3158	72	65	182	675	703	23.0	11.4	2.9	1.4	25.5	20.8
Norway	3422	101	149	112	870	325	13.7	13.0	1.4	2.8	18.9	54.7
Oman	3266	88	89	161	1008	346	19.4	11.6	1.5	2.1	22.1	31.3
Pakistan	2738	72	82	40	795	259	10.4	8.1	0.9	1.7	10.2	32.0
Panama	2900	76	91	62	389	517	18.8	10.2	2.4	1.8	26.0	31.1
Paraguay	3021	73	111	139	584	598	16.6	11.4	2.6	1.9	23.1	37.3
Peru	2609	71	54	133	882	739	23.0	10.0	2.8	1.9	26.7	18.8
Philippines	2710	63	56	109	487	375	19.9	9.1	2.6	1.4	25.1	24.7
Poland	3565	93	142	99	1005	312	12.6	11.0	1.7	2.3	20.5	53.0
Portugal	3648	104	165	153	1169	375	16.9	12.8	1.8	2.5	22.4	55.2
Republic of Korea	3176	87	115	164	1095	454	24.1	12.7	2.3	2.0	22.3	36.5
Republic of Moldova	2258	61	84	59	531	189	11.1	8.9	1.4	1.9	15.0	24.1
Romania	3601	105	120	152	1166	417	17.9	12.8	1.8	2.8	21.1	37.6
Russian Federation	3347	94	117	103	811	314	13.5	10.6	1.4	2.0	19.0	36.7
Rwanda	2357	63	22	206	1107	864	20.1	10.0	2.2	0.7	18.1	6.5
Saint Kitts and Nevis	2031	63	48	76	295	132	7.7	7.0	0.7	1.1	15.8	22.5
Saint Lucia	2780	93	79	83	567	316	11.6	9.0	1.4	1.4	21.8	36.7
Saint Vincent and the Grenadines	2796	89	53	145	540	370	13.7	9.1	1.6	1.3	22.6	18.2
Samoa	3412	86	176	152	529	337	18.5	11.3	1.2	1.1	23.4	111.4
Sao Tome and Principe	2523	55	82	74	143	304	14.5	7.7	1.0	0.6	14.2	63.0
Saudi Arabia	3118	84	104	85	1090	858	24.4	10.3	2.9	2.5	32.4	34.5
Senegal	2718	65	75	44	451	436	19.4	9.9	2.5	1.4	25.5	20.0
Sierra Leone	2358	51	55	115	812	383	15.9	7.7	2.1	0.8	19.2	19.0
Slovakia	3200	78	128	65	565	253	10.4	9.0	1.2	2.1	14.9	41.6
Slovenia	3308	95	139	96	934	305	14.7	12.1	1.5	2.8	19.0	51.7
Solomon Islands	2323	53	53	146	1605	623	19.5	8.0	2.3	0.8	19.0	35.5
South Africa	3245	89	89	47	511	668	22.7	13.0	3.1	2.3	33.6	24.4
Spain	3376	96	169	100	745	334	14.3	11.6	1.6	2.1	20.0	46.2
Sri Lanka	2796	61	61	58	375	289	21.1	9.1	1.8	1.0	17.6	42.9
Suriname	2804	65	84	110	431	548	17.8	8.0	2.3	1.5	25.5	28.6
Sweden	3487	100	164	122	1335	311	12.5	12.6	1.5	3.2	18.5	64.1
Switzerland	3645	93	165	116	1145	305	12.6	11.4	1.3	3.0	16.2	59.3
Tajikistan	2200	68	50	96	526	297	14.9	9.4	1.4	1.3	15.9	13.0
Thailand	2786	61	58	120	625	245	21.5	8.8	2.0	1.2	19.6	21.4
The former Yugoslav Republic of Macedonia	3040	80	113	150	1097	396	16.8	10.1	1.5	2.6	16.6	31.1
Togo	2612	62	49	104	345	489	17.6	10.7	2.5	1.0	21.9	17.0
Trinidad and Tobago	3209	90	104	102	490	913	22.7	10.0	2.9	2.5	33.3	41.6
Tunisia	3601	104	100	191	1070	533	19.4	11.0	1.4	2.1	17.5	30.2
Turkey	3956	112	129	174	1178	582	20.4	13.1	1.8	2.7	19.2	38.4
Turkmenistan	2836	93	74	86	540	1442	27.7	10.6	4.2	3.8	39.2	25.8
Uganda	2314	56	52	103	835	586	15.4	9.1	1.9	1.0	16.9	13.9
Ukraine	3117	85	102	127	828	341	14.2	10.3	1.5	2.0	18.7	30.3
United Arab Emirates	3632	115	108	94	621	594	22.5	14.1	1.9	2.2	22.8	32.5
United Kingdom	3282	94	134	117	792	840	20.8	11.5	2.9	3.1	29.9	44.0
United Republic of Tanzania	2457	61	54	109	774	596	18.0	10.4	2.3	1.1	20.0	17.0
United States of America	3869	105	189	103	742	752	20.0	14.2	2.6	3.4	32.2	63.7
Uruguay	3384	101	114	83	800	1011	23.6	12.9	3.3	3.4	34.6	41.6
Uzbekistan	2758	85	72	177	988	393	15.3	9.7	1.2	2.1	13.9	23.7
Vanuatu	2938	70	122	113	476	443	20.7	10.0	1.7	0.8	20.5	83.0
Venezuela (Bolivarian Republic of)	3040	82	104	87	607	597	18.3	11.2	2.5	2.2	27.8	34.1
Viet Nam	2823	80	71	155	885	451	27.4	11.7	3.1	1.7	27.1	24.3
Yemen	2190	58	44	46	465	891	19.4	6.7	2.7	2.0	25.8	14.5
Zambia	2144	57	47	60	373	286	15.5	10.5	1.9	1.1	17.8	10.8
Zimbabwe	2295	52	60	26	245	326	14.4	9.3	2.0	1.2	19.2	14.8
